# Structural and mechanistic insights into the UBR4-KCMF1-Calmodulin complex

**DOI:** 10.1093/procel/pwag036

**Published:** 2026-05-24

**Authors:** Ziwei Hu, Zhiheng Liu, Jiali Xu, Baotong Zhang, Renhong Yan

**Affiliations:** SUSTech Homeostatic Medicine Institute, School of Medicine, Southern University of Science and Technology, Shenzhen 518005, China; Key University Laboratory of Metabolism and Health of Guangdong, Institute for Biological Electron Microscopy, Southern University of Science and Technology, Shenzhen 518005, China; SUSTech Homeostatic Medicine Institute, School of Medicine, Southern University of Science and Technology, Shenzhen 518005, China; Key University Laboratory of Metabolism and Health of Guangdong, Institute for Biological Electron Microscopy, Southern University of Science and Technology, Shenzhen 518005, China; SUSTech Homeostatic Medicine Institute, School of Medicine, Southern University of Science and Technology, Shenzhen 518005, China; Key University Laboratory of Metabolism and Health of Guangdong, Institute for Biological Electron Microscopy, Southern University of Science and Technology, Shenzhen 518005, China; SUSTech Homeostatic Medicine Institute, School of Medicine, Southern University of Science and Technology, Shenzhen 518005, China; Key University Laboratory of Metabolism and Health of Guangdong, Institute for Biological Electron Microscopy, Southern University of Science and Technology, Shenzhen 518005, China; SUSTech Homeostatic Medicine Institute, School of Medicine, Southern University of Science and Technology, Shenzhen 518005, China; Key University Laboratory of Metabolism and Health of Guangdong, Institute for Biological Electron Microscopy, Southern University of Science and Technology, Shenzhen 518005, China

**Dear Editor,**

Protein quality control is essential for cellular homeostasis (Costa-Mattioli and Walter, 2020; Ng et al., 2021). Misfolded protein accumulation underlies Parkinson’s disease and cancer progression (Kwan et al., 2023; Siderowf et al., 2023). To prevent such damage, cells rely on dedicated protein surveillance and degradation pathways. The ubiquitin-proteasome system depends not only on the E1-E2-E3 cascade, but also on accessory factors that mediate substrate recognition and ubiquitin chain extension. UBR4 has recently emerged as a core scaffold for a stress-responsive complex that forms a complex with KCMF1 and calmodulin (CaM) (Hong et al., 2015; Singh et al., 2023; Varland et al., 2023). The human UBR4-KCMF1-CaM (UKC) complex functions to suppress the integrated stress response triggered by mitochondrial import stress (Cervia et al., 2023; Haakonsen et al., 2024). Although recent studies have defined the ring architecture of UBR4-containing assemblies (Grabarczyk et al., 2025; Yang et al., 2025), the contributions of KCMF1 to complex assembly and catalysis, the mechanisms by which CaM regulates enzymatic activity, and the relationship between these features and disease-relevant phenotypes require further investigation.

We first investigated the biological importance of the UKC complex in prostate cancer. Depletion of either UBR4 or KCMF1 significantly inhibited DU 145 xenograft tumor growth (Fig. 1A and 1B). Consistently, KCMF1 or UBR4 knockdown impared cell viability under mitochondrial stress (oligomycin treatment) and sensitized the cells to most chemotherapeutic agents including docetaxel, cisplatin, and 5-fluorouracil (Fig. S1). This finding suggests that UBR4 and KCMF1 plays a critical role in cancer cells for stress adaptation.

**Figure 1.**
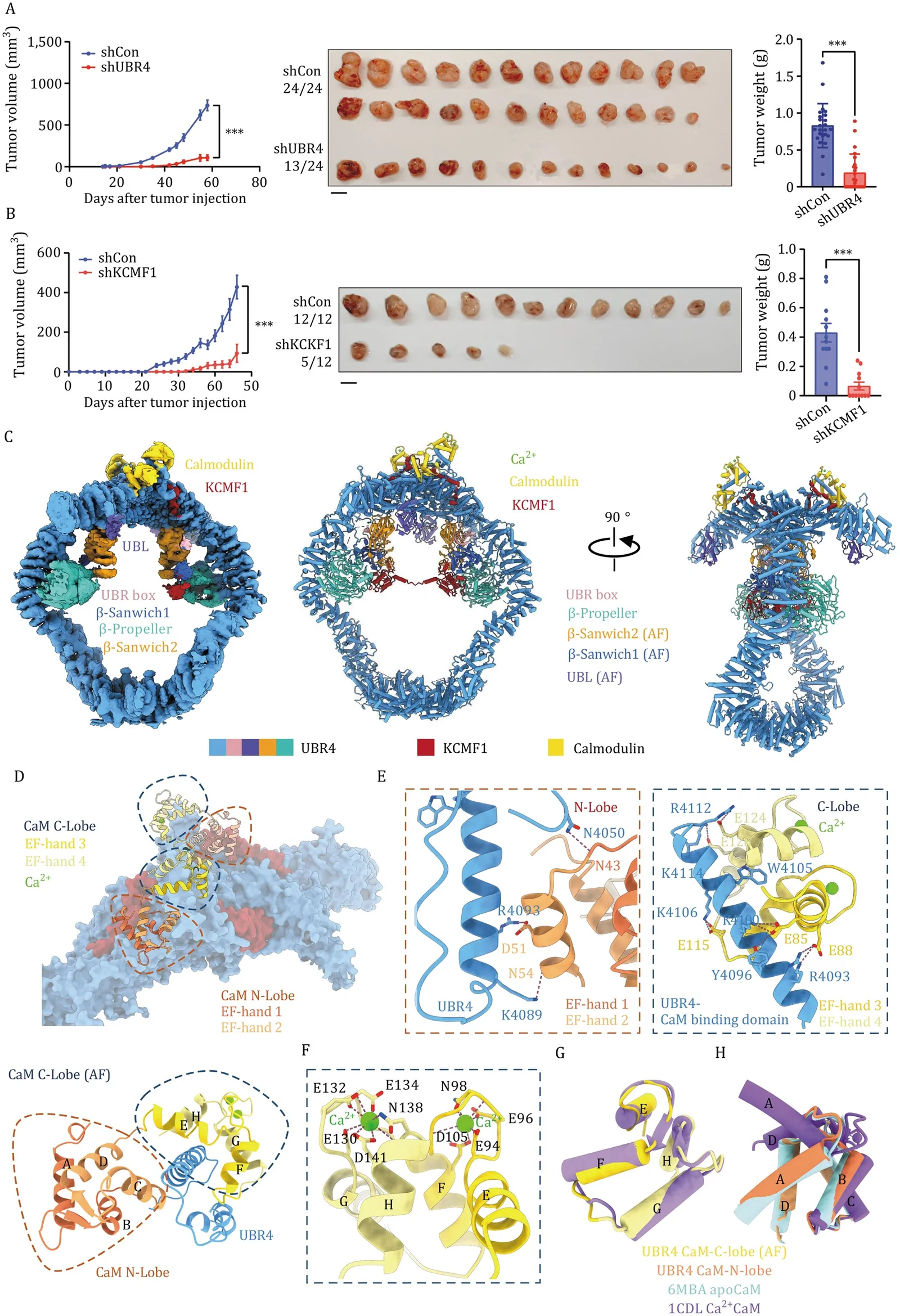
**Cancer biological functional characterization and overall structure of the UBR4-KCMF1-CaM (UKC) complex**. (A and B) Knockdown of UBR4 (A) or KCMF1 (B) suppressed tumor formation of prostate cancer DU 145 cells in a mouse xenograft model, as shown by tumor growth curves (left), images of excised tumors (middle), and tumor weights (right). In panel (A), *n *= 24 tumors; in panel (B), *n *= 12 tumors. ***, *P *< 0.001 by two-way ANOVA for tumor growth curves and two-tailed Student’s t-tests for tumor weights. (C) Cryo-EM reconstruction of the UKC complex at 3.89 Å resolution, shown as an electron density map (left) and the corresponding fitted atomic model in two different orientations (middle and right). Structural domains are color-coded as follows: UBR4 scaffold, cornflower blue; UBL, purple; β-sandwich 1, royal blue; β-sandwich 2, orange; β-propeller, turquoise; UBR box, pink; KCMF1, red; and calmodulin (CaM), gold. AF, AlphaFold-predicted. (D) Topology of CaM upon UBR4 binding. The upper panel shows an overview of the fitted model of UBR4-bound CaM, with putative Ca^2+^ ions shown in green. The lower panel shows the corresponding structural model, highlighting the CaM C-lobe and N-lobe with blue and orange dashed outlines, respectively. The N-lobe adopts a semi-open conformation, whereas the C-lobe adopts an open Ca^2+^-bound-like conformation. (E) Detailed views of the interaction interfaces between UBR4 and the CaM N-lobe (left) and C-lobe (right), with key residues indicated. (F) Detailed view of Ca^2+^-binding sites in the CaM C-lobe. (G) Structural comparison of the UBR4-bound CaM C-lobe (yellow; AF-guided model) with Ca^2+^-bound calmodulin (purple; PDB: 1CDL). (H) Structural comparison of the UBR4-bound CaM N-lobe (orange) with apo-calmodulin (cyan; PDB: 6MBA), with Ca^2+^-bound calmodulin (purple; PDB: 1CDL) shown for reference.

To investigate the structural basis of this functional asymmetry, we purified the human UKC complex and determined its cryo-EM structure at 3.89 Å resolution. The reconstruction revealed a toroidal architecture in which KCMF1 sits within the UBR4 scaffold ring (Figs. 1C and S2–6). Through focused cryo-EM processing and local refinement, we resolved the N-lobe, C-lobe, and propeller region, together with additional densities assigned to KCMF1 (Fig. S3–9). Endogenous calmodulin (CaM) was identified as a co-purified component (Fig. S6 and Table S1), with reconstruction and model statistics summarized in Table S2. We next characterized the enzymatic activity of this assembly. *In vitro* assays showed that the UKC complex preferentially employs UBE2A rather than UBE2D3 as its E2 enzyme (Fig. S10A). *In vitro* ubiquitination assays showed that efficient modification required a substrate carrying a pre-attached ubiquitin moiety, as the ubiquitin-fused mitochondrial targeting sequence of the mitochondrial acyl-CoA thioesterase ACOT9 (ACOT9-MTS-Ub) was ubiquitinated much more effectively than the corresponding unmodified ACOT9-MTS substrate (Fig. S10B). Increasing UKC concentrations further enhanced ACOT9-MTS-Ub ubiquitination in a dose-dependent manner (Fig. S10C).

Together, the toroidal C-terminal scaffold and the additional contacts within the N-terminal scaffold indicate that UBR4 is more than a passive structural scaffold. Instead, UBR4 forms an extended platform that stabilizes the ring and helps shape the reaction space (Fig. S7–9). Within this assembly, calmodulin binds in an asymmetric manner (Fig. 1D). The C-terminal lobe adopts an open Ca^2+^-bound-like conformation and engages UBR4 through EF-hands 3 and 4, whereas the N-terminal lobe remains semi-closed (Fig. 1D–H). Because the CaM C-lobe appears relatively flexible and its local density does not allow unambiguous assignment of all Ca^2+^ ions, these ions are shown as putative, model-guided placements. The C-lobe was modeled based on the Ca^2+^-bound calmodulin-target complex (PDB: 1CDL) and fitted into the observed density with AlphaFold-assisted guidance (Meador et al., 1992). Moreover, EGTA chelation reduced UKC-dependent ubiquitination (Fig. S10D). We next examined additional MTS-containing substrates derived from COX8A, COQ9, and NIPSNAP1. Like ACOT9-MTS, these substrates were efficiently modified only in their ubiquitin-fused forms (Fig. S11A), and substrate titration further confirmed UKC-mediated ubiquitination of their MTS-Ub forms (Fig. S11B–D). These results support an E4-like role for UKC in extending pre-existing ubiquitin signals rather than efficiently catalyzing *de novo* substrate priming.

KCMF1 plays a central role in both complex assembly and catalytic activation. Structural analysis showed that the N-terminal ZZ-DZB module and the C-terminal helical region contribute differently to UBR4 binding (Fig. 2A). Biolayer interferometry experiments confirmed that removing either region reduced the binding affinity for UBR4 (Fig. 2B). Cellular assays showed that overexpressing UBR4 induced dose-dependent degradation of ACLY, DELE1, and HRI. This activity required an intact interface between UBR4 and KCMF1 (Fig. 2C–E). In reconstituted ubiquitination assays, KCMF1 substantially enhanced UBR4-mediated polyubiquitination (Fig. 2F). Mutating the conserved ZZ-DZB residues Asp29 and Asp31 to arginine severely impaired ubiquitination of mitochondrial targeting sequence-containing substrates (Fig. 2G). Biochemical studies further revealed that UBR4 alone could not be purified as a stable homogeneous protein and required co-expression with KCMF1 (Fig. S12). This indicates that KCMF1 is essential for maintaining the structural integrity of the complex. Systematic mutagenesis of the ZZ-DZB region produced variants with graded defects in UBR4 binding and corresponding reductions in ubiquitination activity (Fig. S13A–C). These findings suggest that the ZZ-DZB region recognizes and binds pre-ubiquitinated substrates, while the C-terminal helix maintains the structural scaffold necessary for efficient chain elongation.

**Figure 2.**
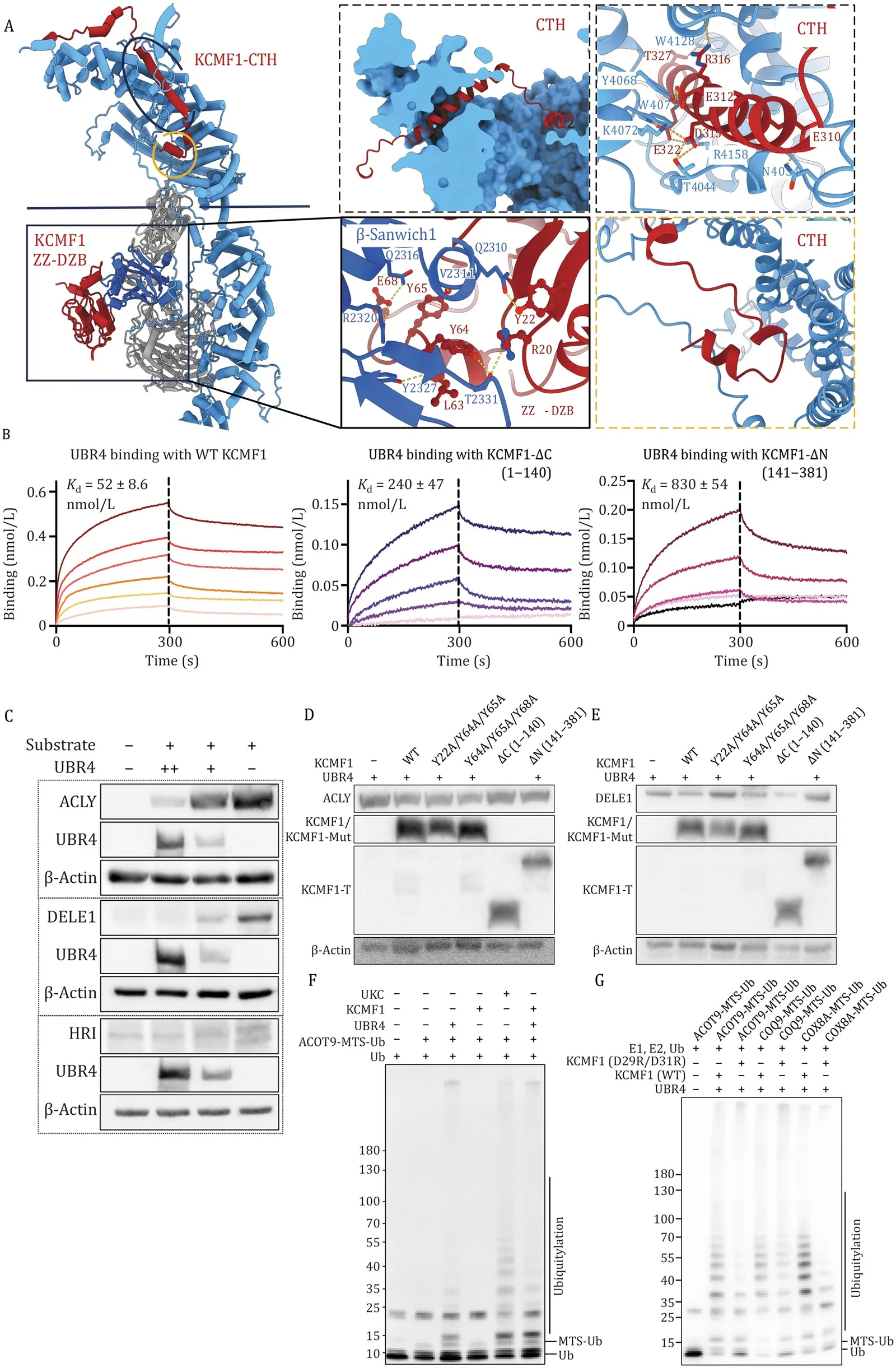
**KCMF1 mediates UBR4 complex assembly and promotes substrate ubiquitination**. (A) Structural view of the KCMF1-UBR4 interface. UBR4 is shown in cornflower blue and KCMF1 in red. Insets highlight the KCMF1 C-terminal helix (CTH) docked on the UBR4 scaffold and the ZZ-DZB region contacting the UBR4 β-sandwich 1 domain, with key interface residues indicated. (B) Biolayer interferometry analysis of binding between UBR4 and wild-type (WT) KCMF1, KCMF1-ΔC (1–140), or KCMF1-ΔN (141–381). Dissociation constants (K_d_) are indicated. (C) Immunoblot analysis in C4-2B cells showing dose-dependent degradation of ACLY, DELE1, and HRI upon UBR4 overexpression. (D and E) Immunoblot analysis of the UBR4 substrates ACLY (D) and DELE1 (E) in C4-2B cells expressing UBR4 together with WT or mutant/truncated KCMF1. KCMF1 mutations or truncations impaired UBR4-induced substrate degradation. KCMF1/KCMF1-Mut denotes full-length WT or point-mutant KCMF1, whereas KCMF1-T denotes truncated KCMF1 variants. (F) *In vitro* ubiquitination assays showing that KCMF1 enhances UBR4-mediated ubiquitination of the ACOT9-MTS-Ub substrate. Reactions contained the indicated combinations of UKC, recombinant KCMF1, UBR4, ACOT9-MTS-Ub and Ub. (G) *In vitro* ubiquitination assays comparing WT KCMF1 and the KCMF1 D29R/D31R mutant in UBR4-containing reactions with the indicated MTS-Ub substrates, including ACOT9-MTS-Ub, COQ9-MTS-Ub, and COX8A-MTS-Ub. UKC, UBR4-KCMF1-CaM complex; CTH, C-terminal helix; MTS-Ub, mitochondrial targeting sequence-tagged ubiquitin; Ub, ubiquitin; D29/D31, Asp29/Asp31.

Our structure also provides a framework for understanding disease-associated variation in UBR4. Several reported UBR4 variants linked to neurological disease or cancer near the CaM-binding region (Fig. S14). Among them, R4111H lies close to the calcium-responsive interface and may alter local regulatory interactions (Choi et al., 2017; Conroy et al., 2014; Gaudio et al., 2023). These observations raise the possibility that defective calcium-dependent control of the UBR4-KCMF1 complex contributes to both neuronal dysfunction and tumor biology.

While this work was being completed, independent studies reported the ring architecture of UBR4 and its role within the silencing factor of the integrated stress response (SIFI) machinery (Grabarczyk et al., 2025; Yang et al., 2025). Our data extend this framework by defining a disease-relevant functional division within the complex. In this model, UBR4 mainly serves as a scaffolded E4 ligase that elongates pre-existing ubiquitin signals, whereas KCMF1 plays a more direct role in substrate recognition and processing. This organization may explain why KCMF1 depletion produces a stronger phenotype than UBR4 depletion in stress-sensitivity assays. The distinct contributions of the two subunits to proteostasis control further suggest that targeting the KCMF1-substrate interface may offer a more selective therapeutic strategy than global inhibition of UBR4 in prostate cancer.

This study also has some limitations. Although our data support an E4-like role for the UKC complex in chain elongation, they do not exclude the possibility that UBR4 may also exert E3-like functions in other settings. In addition, the mechanism of calcium-dependent regulation still requires direct kinetic analysis, and the key substrates responsible for the tumor growth phenotypes remain to be defined *in vivo*.

In summary, our work reveals the structural basis of UBR4-KCMF1-CaM assembly and establishes KCMF1 as a critical node linking ubiquitin signaling to cancer cell fitness and chemotherapy response. These insights provide a rationale for developing targeted therapies against stress-adaptation pathways in prostate cancer and neurodegenerative disorders.

## Supplementary Material

pwag036_Supplementary_Data

## Footnotes

We thank the Cryo-EM Facility of Southern University of Science and Technology (SUSTech) for facility support. We thank Yuanzhu Gao, Shuman Xu, Lei Zhang, and Peiyao Li at the Cryo-EM Center of SUSTech for technical support. Renhong Yan is an investigator of the SUSTech Institute for Biological Electron Microscopy. We thank Zhenyuan Liu for technical support and Drs. Yong Tae Kwon and Min Ju Lee for providing UBR4 plasmids.

This work was supported by the Shenzhen Medical Research Fund (E24010010 and E24010012 to R.Y.), the National Natural Science Foundation of China (32422039 and 82202517 to R.Y., 324B2040 to Z. H., 82273079 and 82473084 to B.Z.), the Shenzhen Excellent Science and Technology Innovation Talent Training Program (RCYX20231211090342048 to R.Y.), the Science and Technology Innovation Commission of Shenzhen Municipality (JSGG20220226085550001 to R.Y.), and the Major Talent Recruitment Program of Guangdong Province (2021QN02Y167 to R.Y. and 2021QN02Y875 to B.Z.).

R.Y. and B.Z. conceived the project and designed the experiments. Z.H. and Z.L. carried out protein purification, cryo-EM analyses, BLI assay and biochemical experiments. J.X. performed cell-based and animal experiments. R.Y., B.Z., Z.H., Z.L., and J.X. wrote the manuscript. All authors read and approved the final manuscript.

The overall structure of the human UBR4-KCMF1-calmodulin complex has been deposited in the Protein Data Bank under accession code 9XTJ, and the corresponding cryo-EM map has been deposited in the Electron Microscopy Data Bank under accession code EMD-67234. The focused reconstructions of the N-terminal lobe and C-terminal lobe have been deposited in the Protein Data Bank under accession codes 9XKL and 9XTI, respectively, with the corresponding EMDB accession codes EMD-66968 and EMD-67233. The focused cryo-EM map of the propeller domain of human UBR4 has been deposited in the EMDB under accession code EMD-67245. Correspondence and requests for materials should be addressed to R.Y. (yanrh@sustech.edu.cn).

Ziwei Hu, Zhiheng Liu, Jiali Xu, Baotong Zhang, and Renhong Yan declare no competing interests.

All animal procedures were performed in accordance with institutional and national guidelines for the care and use of laboratory animals.
