# Supplementary material for: Structural and mechanistic insights into the UBR4-KCMF1-Calmodulin complex
Source: Protein Cell. 2026 May 24;17(11):1084–8. doi: 10.1093/procel/pwag036 (PMC13721120; doi:10.1093/procel/pwag036)
Supplement: Supplementary file 1 [file pwag036_supplementary_data.pdf]

Supplementary Materials for

Structural and mechanistic insights into the UBR4-KCMF1-Calmodulin complex

Ziwei Hu[1, 2, #], Zhiheng Liu[1, 2, #], Jiali Xu[1, 2, #], Baotong Zhang[1, 2*], Renhong Yan[1, 2*]

[1] SUSTech Homeostatic Medicine Institute, School of Medicine, Southern University of Science and Technology, Shenzhen, Guangdong Province, China.
[2] Key University Laboratory of Metabolism and Health of Guangdong, Institute for Biological Electron Microscopy, Southern University of Science and Technology, Shenzhen, Guangdong Province, China.
[#] These authors contributed equally to this work.
* To whom correspondence should be addressed: yanrh@sustech.edu.cn (R.Y.) and zhangbt@sustech.edu.cn (B.Z.)

This document includes:

Materials and Methods
Supplementary Figures S1 to S14
Supplementary Tables S1 and S3

Materials and Methods

Cell culture

Expi293F cell lines were obtained from ATCC and maintained in Dulbecco's modified Eagle's medium (DMEM) supplemented with 10% fetal bovine serum (FBS) and 100 U/mL penicillin and streptomycin. C4-2B cell lines were obtained from ATCC and maintained in RPMI 1640 supplemented with 10% FBS and 100 U/mL penicillin and streptomycin. Cells were routinely cultured at 37 °C with 5% CO_2.

Knockdown of UBR4 and KCMF1 in C4-2B cell lines

Stable knockdown of UBR4 or KCMF1 was achieved using a lentiviral shRNA system based on the pLKO.1 vector according to the protocol from The RNAi Consortium (TRC). shRNA oligos targeting UBR4 or KCMF1 were designed and synthesized as follows: UBR4 shRNA,5'-CCGGGCCGCTGATGAGGGATATAAACTCGAGTTTATATCCCTCATCAGCGGCTTTTTG-3' and 5'-CCGGGCCGCTGATGAGGGATATAAACTCGAGTTTATATCCCTCATCAGCGGCTTTTTG-3'; KCMF1 shRNA, 5'-CCGGGTCGCAGATATAAGTGTTTAACTCGAGTTAAACACTTATATCTGCGACTTTTTG-3' and 5'-AATTCAAAAAGTCGCAGATATAAGTGTTTAACTCGAGTTAAACACTTATATCTGCGAC-3'. Complementary oligos were annealed and cloned into the AgeI and EcoRI sites of the pLKO.1-puro vector (#10878, Addgene). Lentiviruses were generated following the standard lentiviral packaging protocol described by Addgene. C4-2B cells infected with lentiviruses were selected in medium containing puromycin (1 μg/mL) for 72-96 h before subsequent experiments.

Cell proliferation

Cytotoxicity was evaluated with water-soluble tetrazolium salt in the Cell Counting Kit-8 (MA0225-1, Meilun Bio). Approximately 5×10^3 cells were seeded in 96-well plates. After 24, 48, 72 and 96 hours, CCK-8 was added to each well, and the plates were incubated for 1 hour,

followed by absorbance measurement at 450 nm using the Synergy HTX multi-mode reader (BioTek).

Cytotoxicity assay

Cytotoxicity was evaluated with water-soluble tetrazolium salt in the Cell Counting Kit-8 (MA0225-1, Meilun Bio). Approximately 3×10^3 cells were seeded in 96-well plates for DTX treatment. After 48 hours of DTX treatment, CCK-8 was added to each well, and the plates were incubated for 2 hours, followed by absorbance measurement at 450 nm using the Synergy HTX multi-mode reader (BioTek).

Quantitative real-time reverse transcription-PCR

Total RNA of cells was extracted using RNA-easy Isolation Reagent (R701, Vazyme), and cDNA was synthesized using ExonScript RT Mix (with dsDNase) (TQ-BG-070, Techisun) according to the manufacturer's instructions. mRNA levels were determined using the 7500 Fast Real-Time PCR system (Applied Biosystems, Carlsbad, CA, USA) according to the manufacturer's instructions. Data were normalized to GAPDH mRNA levels for analysis. Experiments were repeated three times, and the fold difference in gene expression in cells transfected with UBR4 or KCMF1 siRNAs compared with control cells was calculated using the comparative Ct method. The following oligonucleotides were used: 5'-TCCTACTCCGCCTTCGAGATG-3' and 5'-CTGAAGTTGGTTCCGGGGAAT-3' for UBR4, 5'-TCGAGGTCGCAGATATAAGTGT-3' and 5'-CCGTATAGCCCATTTTTCCACAA-3' for KCMF1, 5'-ACAACTTTGGTATCGTGGAAGG-3' and 5'-GCCATCACGCCACAGTTTC-3' for GAPDH.

Western blotting analysis and immunoprecipitation assay

Sub-confluent cells were scraped into the culture medium, collected, and washed with cold phosphate-buffered saline (PBS). Cell pellets were lysed in 2× Laemmli sample buffer (Bio-Rad, Hercules, CA, USA) and heated at 98 °C for 5 min. Whole-cell lysates were separated by SDS-PAGE and transferred onto nitrocellulose membranes (Bio-Rad, Hercules, CA, USA). Membranes were blocked with 5% non-fat milk in TBS buffer (pH 8.0) containing 0.1% Tween-20 and incubated overnight at 4 °C with primary antibodies against UBR4 (NBP1-28730, Novus Biologicals), Bcl-2 (15071S, Cell Signaling Technology), β-actin (66009-1-1g, Proteintech), HA

tag (PTM-5389, PTM Bio), FLAG tag (PTM-6075, PTM Bio), and MYC tag (PTM-5169, PTM Bio). Membranes were then washed three times for 5 min each with TBS buffer (pH 8.0) containing 0.1% Tween-20 and incubated with HRP-conjugated secondary antibodies, including anti-rabbit IgG (7074, Cell Signaling Technology) and anti-mouse IgG (7076, Cell Signaling Technology), for 1 h at room temperature. After three additional washes in TBS buffer (pH 8.0) containing 0.1% Tween-20, signals were detected using an ECL detection system (P0018AM, Beyotime).

Statistical analysis

Data are expressed as means ± standard deviation from three or more independent experiments. Statistical analysis was performed using Student's t test.

Protein preparation

The cDNAs encoding full-length UBR4 (1-5183 aa, accession number: AF348492) and KCMF1 (1-381 aa, accession number: AB083199.1) were subcloned into pCAG vectors. For the substrate proteins, the cDNAs encoding full-length human ACLY (1-1101 aa) and HRI (1-615 aa) were similarly cloned into mammalian expression vectors for use in subsequent functional assays. The UBR4 construct was generated with an N-terminal FLAG tag, whereas the KCMF1 construct was generated with a C-terminal 10× His tag using standard PCR-based cloning strategies. Recombinant protein expression was performed in HEK293F mammalian cells cultured at 37 °C in a Multitron-Pro shaker (Infors) at 130 rpm in an atmosphere containing 5% CO_2. When the cell density reached approximately 2.0×10^6 cells/mL, cells were transiently transfected with plasmids prepared using the GoldHi EndoFree Plasmid Maxi Kit (CWBIO). For co-expression of the UBR4-KCMF1 complex, 0.75 mg of each UBR4 and KCMF1 maxi-plasmid was mixed with 3 mg of polyethyleneimine (PEI, Polysciences) in 50 mL of fresh medium, incubated for 20 minutes, and then added to the cell culture. The cells were incubated for 60 h after transfection.

For protein purification, harvested cells were resuspended in lysis buffer consisting of 25 mM HEPES, pH 7.5, 150 mM NaCl, and a protease inhibitor cocktail. The cocktail contained aprotinin at 1.3 μg/mL, pepstatin at 0.7 μg/mL, and leupeptin at 5 μg/mL. Lysis was performed by sonication at 4°C, followed by centrifugation at 16,000× g for 50 minutes to remove cell debris. The supernatant was incubated with anti-FLAG M2 affinity resin (Sigma), washed with 50 mL of

buffer (25 mM HEPES, pH 7.5, 150 mM NaCl), and eluted using the same buffer supplemented with 0.2 mg/mL FLAG peptide. To further purify the protein complex, the eluate was applied to Ni-NTA affinity resin (Qiagen), followed by sequential washes with 20 mL of buffer containing 10 mM imidazole and elution with 10 mL of buffer containing 300 mM imidazole. The purified protein complex was subjected to size-exclusion chromatography (Superose 6 Increase 10/300 GL, GE Healthcare) in buffer (25 mM HEPES, pH 7.5, 150 mM NaCl). Peak fractions were collected, concentrated using a 100 kDa ultrafiltration tube (Merck Millipore), and prepared for electron microscopy (EM) analysis.

Substrate protein preparation

The MTS-coding sequences from ACOT9, NIPSNAP1, COQ9, and COX8A were cloned into the pET21b (+) vector with an N-terminal 6×His-SUMO tag. The MTS sequences used are listed in Table S3. For ubiquitin-fused substrates, a ΔGG ubiquitin moiety was fused to the C terminus of each MTS sequence through a 20-residue glycine-serine linker. The plasmid was transformed into E. coli BL21 (DE3) for expression. The starter culture was diluted into LB supplemented with 100 μg/mL ampicillin and cultured at 37°C until OD_{600} was 0.6-0.8. Protein expression was induced with 0.3 mM IPTG (SJ-MB0016, Sparkjade) and the cultures were incubated overnight at 17 °C. Bacteria were collected by centrifugation at 4000 × g for 15 min. The substrate was purified by Ni-NTA affinity resin (Qiagen). First, bacterial pellets were resuspended in lysis buffer containing 25 mM HEPES, pH 7.5, 400 mM NaCl, and 25 mM imidazole, together with a protease inhibitor mixture comprising aprotinin (Amresco) at 1.3 μg/mL, pepstatin (Amresco) at 0.7 μg/mL, leupeptin (Amresco) at 5 μg/mL, and PMSF (SJ-MX2470, Sparkjade) at 1 mM. Lysis was performed by sonication at 4 °C, followed by centrifugation at 16,000 × g for 50 min to remove debris. The supernatant was loaded onto the Ni-NTA resin three times. The resin was washed with 50 mL wash buffer containing 25 mM HEPES (pH 7.5), 400 mM NaCl and 50 mM imidazole. After overnight cleavage with HRV 3C protease, the protein was eluted with 10 mL of wash buffer. The purified protein was subjected to size-exclusion chromatography (Superose 6 Increase 10/300 GL, GE Healthcare) in buffer (25 mM HEPES, pH 7.5, 300 mM NaCl). Peak fractions were collected and aliquoted, flash frozen and stored at -80 ℃.

***In vitro* ubiquitylation assays**

In vitro ubiquitylation assays were performed in a 10 µL reaction volume. Reactions contained UBA1 (250 nM), UBE2A (2.5 µM), UBE2D3 (2.5 µM), E3 ligase (100 nM), substrate protein (1 µM), 1 mg/mL recombinant human ubiquitin protein (20431ES08, Yeasen), 1ul of 10× reaction buffer (500 mM HEPES, 6 mM DTT). The reaction was started by adding 1 uL 10× ATP-Mg^{2+} (100 mM ATP, 100 mM $MgCl_2$), incubated at 37 °C for 1 h, and was terminated by the addition of EDTA to a final concentration of 20 mM. Ubiquitylation assays were analyzed by 4-20% gradient SDS-PAGE (CWBIO).

Cryo-EM sample preparation and data acquisition

The UBR4-KCMF1 complex was purified and concentrated to approximately 2.5 mg/mL before being applied to the grid for cryo-electron microscopy (cryo-EM) analysis. Small aliquots (3.3 µL) of the protein complex were carefully applied to glow-discharged holey grids with an amorphous alloy film (CryoMatrix, Au R1.2/1.3). After a 3.5-second blotting process, the grids were rapidly plunged into liquid ethane for cryogenic preservation, followed by immersion in liquid nitrogen, using a Vitrobot (Mark IV, Thermo Fisher Scientific). The prepared cryo-EM samples were then loaded into a Titan Krios electron microscope, operated at 300 kV. Cryo-EM data were collected on a Titan Krios electron microscope equipped with a Gatan K3 detector and a GIF Quantum energy filter. Automated image acquisition was performed using AutoEMation, with the energy-filter slit width set to 20 eV. Samples were imaged at a nominal magnification of 105,000× in super-resolution mode, under a range of defocus levels from -1.4 µm to -1.8 µm. Each movie stack was recorded over a total exposure time of 1.71 seconds, with a total electron dose of approximately 50 electrons per Ångström squared (e-/$Å^2$). Following image acquisition, the movie stacks were processed for motion correction using MotionCor2. The images were binned by a factor of 2, resulting in an effective pixel size of 0.827 Å/pixel. Dose-weighting adjustments were applied, and defocus values were accurately estimated using Gctf.

Biolayer interferometry (BLI) assay

BLI was performed using an Octet RED96e instrument (FortéBio) to quantify binding kinetics between biotinylated UBR4 and KCMF1 variants or calmodulin variants. Biotinylation of UBR4 was performed using the Biotinylation Kit (G-MM-IGT-100, Genemore) according to the manufacturer's instructions. Briefly, purified UBR4 was mixed with the 10 mM biotinylation

reagent at a protein-to-reagent molar ratio of 1:3 and incubated at 37 °C for 30 min. Excess free biotinylation reagent was then removed using the desalting column supplied with the kit, and the biotinylated UBR4 was collected for subsequent BLI assays.

Biolayer interferometry assays were performed using streptavidin (SA) biosensors. The biosensors were hydrated in HEPES buffer for 10 min, and biotinylated UBR4 was immobilized at 20 μg/mL for 300 seconds. KCMF1 variants were used as analytes and prepared as a six-point twofold dilution series from 200 to 6.25 nM in kinetics buffer. Binding assays were performed at 25 °C with orbital shaking at 1,000 rpm, including a 300-s association phase followed by a 600-s dissociation phase. Reference sensors subjected to the same assay cycle without UBR4 loading were used for baseline correction. Data were globally fitted to a 1:1 Langmuir binding model using Octet Analysis Studio v12.0 to derive association (K_{on}), dissociation (K_{off}), and equilibrium dissociation constants (K_d).

Data processing

Particle selection from the micrographs was conducted manually, followed by automatic extraction using CryoSPARC v4.4.1 [1, 2]. The initial phase involved a round of 2D classification, after which a subset of high-quality particles was selected for advanced processing. This next step involved 3D reconstruction through Ab-initio Reconstruction, yielding an initial coarse resolution model of the complex. For better resolution refinement, the Heterogeneous Refinement method was utilized. Throughout this process, the current resolution, assessed via Fourier Shell Correlation, along with other diagnostic information, was displayed iteratively. The final resolution was estimated using the gold-standard Fourier shell correlation criterion of 0.143 [3, 4]. Additional details regarding data collection and processing can be found in Supplementary Fig. S3 and Table S2.

Model building and structure refinement

Atomic models of the UBR4-KCMF1 complex were built based on the corresponding cryo-EM maps, using AlphaFold 3 predictions as initial templates. The models were then manually adjusted in Coot (v0.9.8.1) [5], with careful inspection of residue geometry and side-chain placement. To achieve precise refinement, we employed real-space refinement techniques using the Phenix software (v1.20.1-4487) [6]. During this phase, secondary structure and geometric constraints were applied to prevent potential overfitting. The refinement process was carried out in a stepwise

manner to ensure model integrity and minimize overfitting risk. Initially, the model was refined against one of the two independent half maps, in accordance with gold-standard 3D refinement principles. Subsequently, the refined model was validated against the second half map to ensure its accuracy and reliability. Comprehensive statistical data on data collection, 3D reconstruction, and model refinement are provided in Supplementary Fig. S3 and Table S2.

Data availability

The overall structure of the human UBR4-KCMF1-Calmodulin complex has been deposited in the Protein Data Bank under accession code 9XTJ, and the corresponding cryo-EM map has been deposited in the Electron Microscopy Data Bank under accession code EMD-67234. The focused reconstructions of the N-terminal lobe and C-terminal lobe have been deposited in the Protein Data Bank under accession codes 9XKL and 9XTI, respectively, with the corresponding EMDB accession codes EMD-66968 and EMD-67233. The focused cryo-EM map of the propeller domain of human UBR4 has been deposited in the EMDB under accession code EMD-67245. Correspondence and requests for materials should be addressed to R.Y. (yanrh@sustech.edu.cn).

Fig. S1

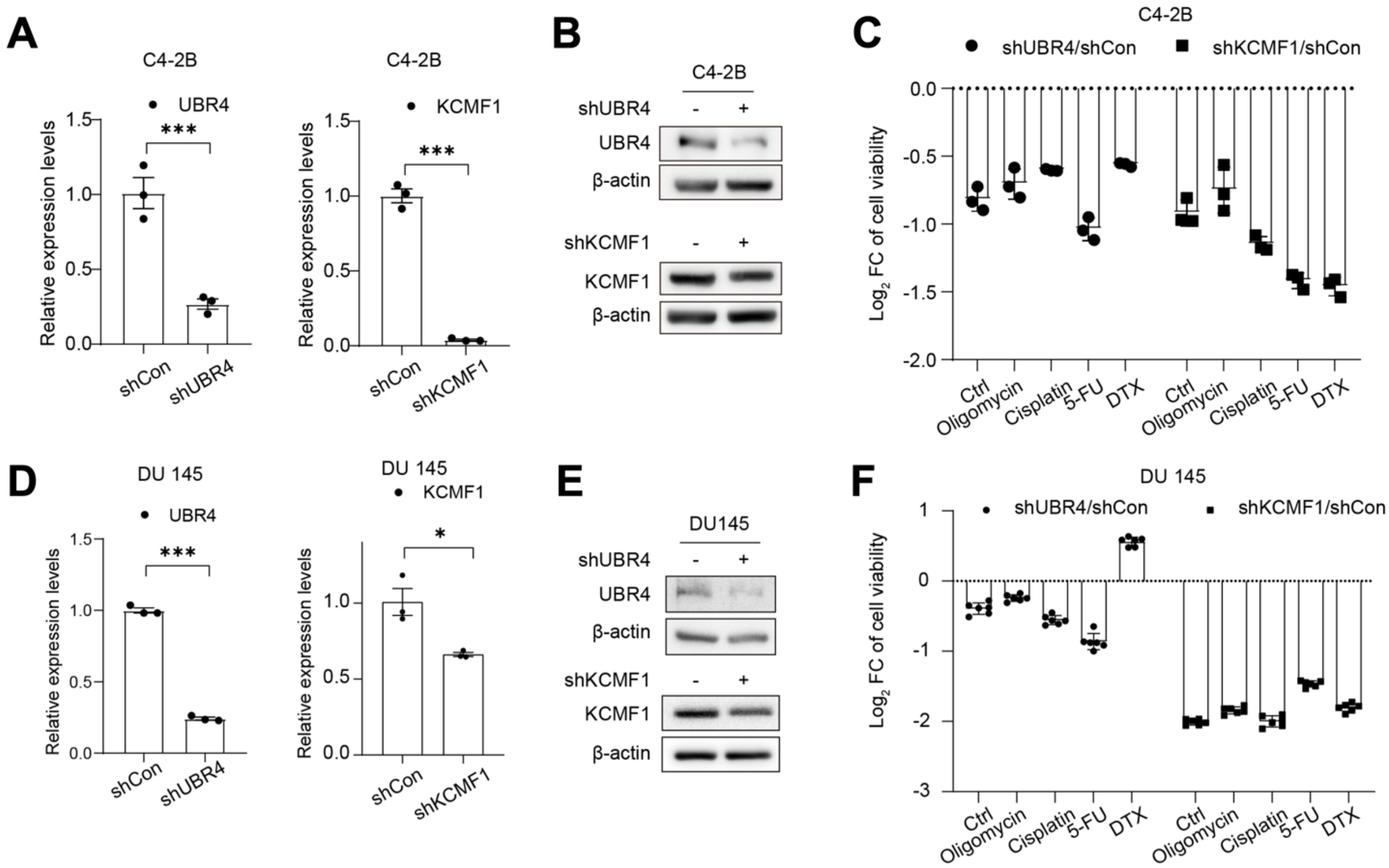

Fig. S1. Efficiency of UBR4 and KCMF1 knockdown and cell viability under chemotherapy-induced stress.

(A, B, D, E) Knockdown efficiency of UBR4 and KCMF1 in C4-2B (A, B) and DU 145 (D, E) cells, assessed by quantitative PCR (A, D) and immunoblotting (B, E). *, P < 0.05; ***, P < 0.001; two-tailed Student's t test. (C, F) Fold change in cell viability after knockdown of UBR4 or KCMF1 under oligomycin-induced mitochondrial stress (3 μM) or treatment with clinically relevant chemotherapeutic agents, including docetaxel (DTX, 10 nM), cisplatin (2 μM), and 5-fluorouracil (5-FU, 2 μM). Data are presented as mean ± SEM from three independent experiments in C4-2B cells (C) or six independent experiments in DU 145 cells (F).

Fig. S2

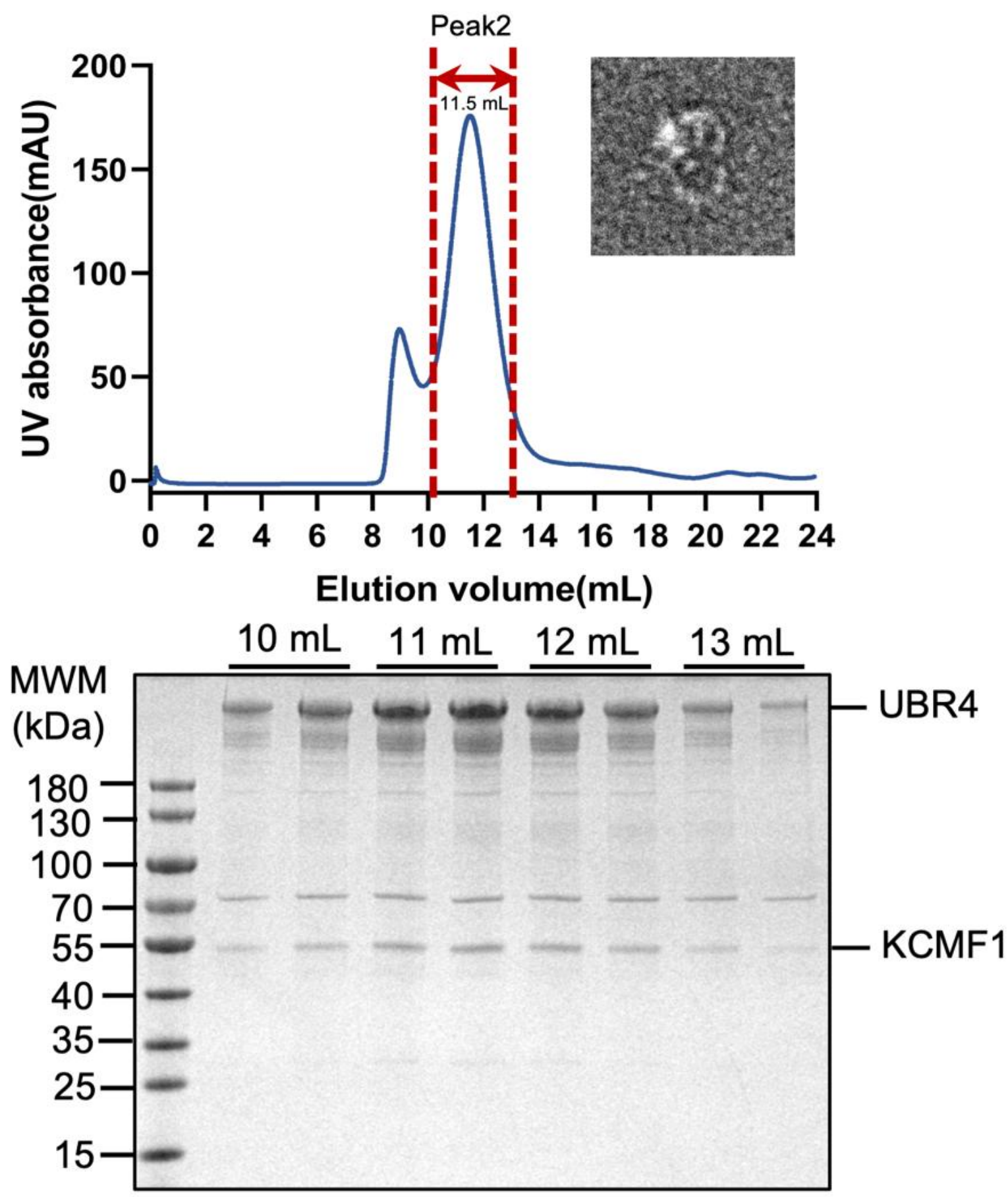

Fig. S2. Size-exclusion chromatography profile of the recombinant UKC complex (theoretical MW ~574 kDa; measured MW, 580 ± 18 kDa), with the corresponding Coomassie-stained SDS-PAGE gel and negative-stain EM 2D class averages (inset; scale bar, 50 Å). MWM, molecular weight marker.

Fig. S3

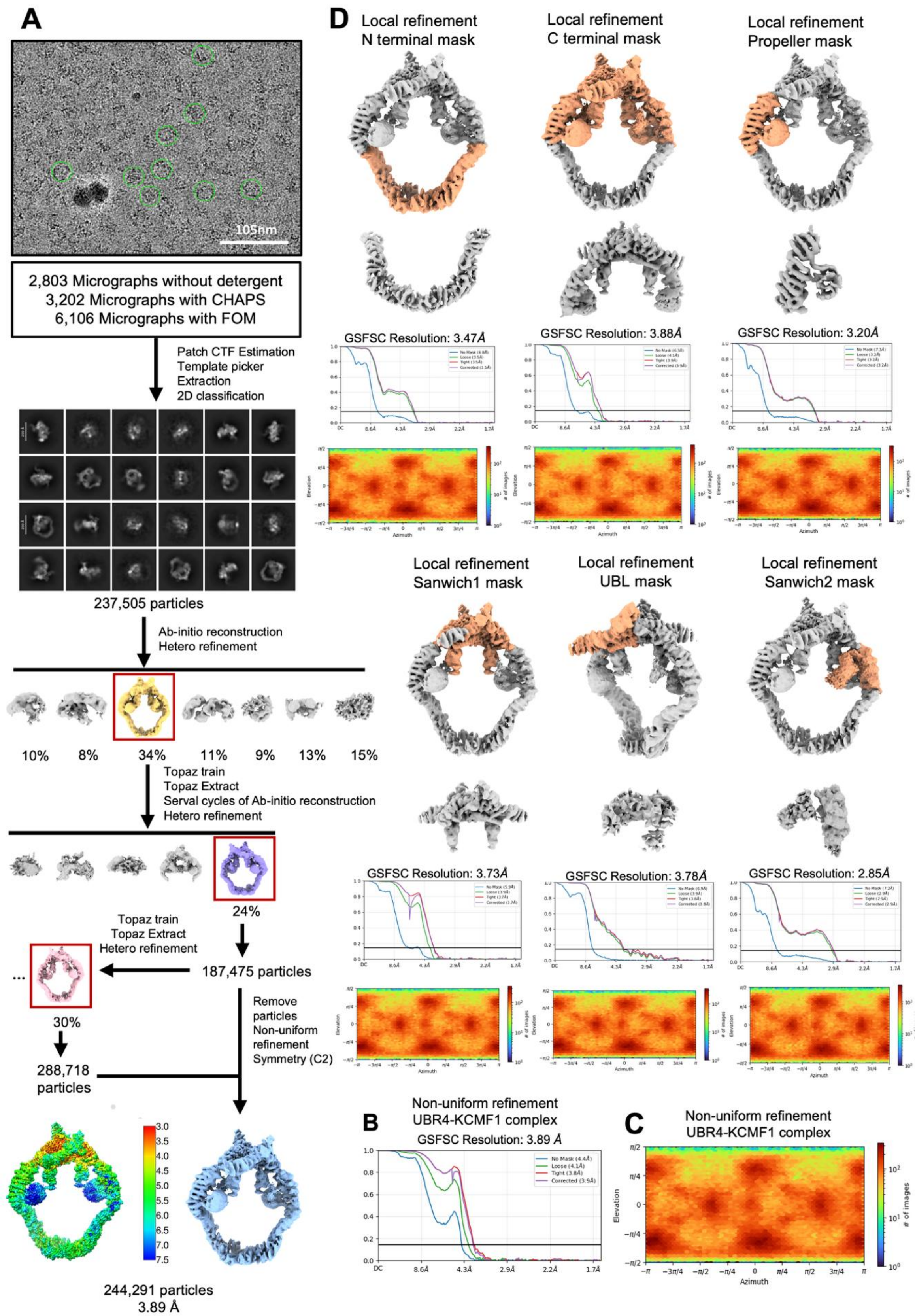
A
105nm
2,803 Micrographs without detergent
3,202 Micrographs with CHAPS
6,106 Micrographs with FOM
Patch CTF Estimation
Template picker
Extraction
2D classification
237,505 particles
Ab-initio reconstruction
Hetero refinement
10%
8%
34%
11%
9%
13%
15%
Topaz train
Topaz Extract
Serval cycles of Ab-initio reconstruction
Hetero refinement
24%
Topaz train
Topaz Extract
Hetero refinement
187,475 particles
30%
288,718 particles
Remove particles
Non-uniform refinement
Symmetry (C2)
244,291 particles
3.89 Å
B
Non-uniform refinement
UBR4-KCMF1 complex
GSFSC Resolution: 3.89 Å
No Mask (4.4Å)
Loose (4.1Å)
Tight (3.8Å)
Corrected (3.9Å)
C
Non-uniform refinement
UBR4-KCMF1 complex
Elevation
Azimuth
# of images
D
Local refinement
N terminal mask
Local refinement
C terminal mask
Local refinement
Propeller mask
GSFSC Resolution: 3.47Å
GSFSC Resolution: 3.88Å
GSFSC Resolution: 3.20Å
Local refinement
Sanwich1 mask
Local refinement
UBL mask
Local refinement
Sanwich2 mask
GSFSC Resolution: 3.73Å
GSFSC Resolution: 3.78Å
GSFSC Resolution: 2.85Å

Fig. S3. Cryo-EM data processing workflow and local refinement of the UKC complex.

(A) Workflow for cryo-EM data processing of the UBR4-KCMF1-CaM complex. (B) Gold-standard Fourier shell correlation curve for the global reconstruction, with an overall resolution of 3.89 Å. (C) Euler angle distribution of the refined particles. (D) Representative focused maps from local refinement, showing improved density for the N-lobe, C-lobe, β-propeller, β-sandwich domains, and calmodulin.

Fig. S4

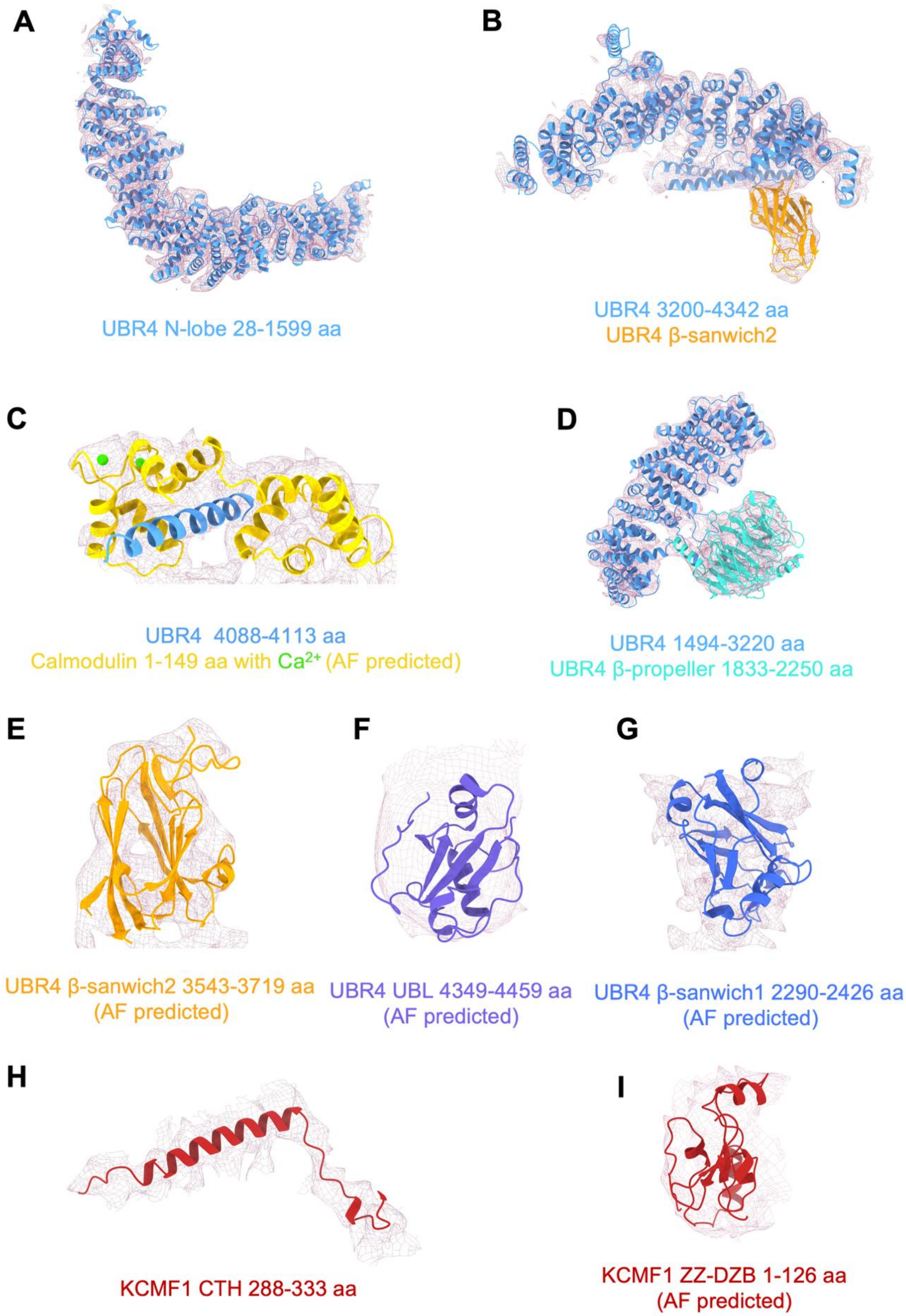
A
UBR4 N-lobe 28-1599 aa
B
UBR4 3200-4342 aa
UBR4 β-sanwich2
C
UBR4 4088-4113 aa
Calmodulin 1-149 aa with Ca^{2+} (AF predicted)
D
UBR4 1494-3220 aa
UBR4 β-propeller 1833-2250 aa
E
UBR4 β-sanwich2 3543-3719 aa
(AF predicted)
F
UBR4 UBL 4349-4459 aa
(AF predicted)
G
UBR4 β-sanwich1 2290-2426 aa
(AF predicted)
H
KCMF1 CTH 288-333 aa
I
KCMF1 ZZ-DZB 1-126 aa
(AF predicted)

Fig. S4. Representative local cryo-EM density views of the UBR4-KCMF1-CaM complex.

(A) Density for the UBR4 N-lobe (residues 28-1599), shown at a threshold of 5σ. (B) Density for the UBR4 region spanning residues 3200-4342, including the β-sandwich 2 domain, shown at a threshold of 5σ. (C) Density for the UBR4 CaM-binding region (residues 4088-4113) together with calmodulin. The calmodulin model, including the putative Ca^{2+} positions, is shown as AF-guided interpretation where indicated. Density is shown at a threshold of 6σ. (D) Density for the UBR4 region spanning residues 1494-3220, including the β-propeller domain, shown at a threshold of 6σ. (E) Density for the UBR4 β-sandwich 2 domain (residues 3543-3719), shown at a threshold of 7σ. The model is labeled AF predicted where the local density is insufficient to support confident map-based modeling. (F) Density for the UBR4 UBL domain (residues 4349-4459), shown at a threshold of 7σ. The model is labeled AF predicted where indicated. (G) Density for the UBR4 β-sandwich 1 domain (residues 2290–2426), shown at a threshold of 7σ. The model is labeled AF predicted where indicated. (H) Density for the KCMF1 C-terminal helix (CTH; residues 288-333), shown at a threshold of 6σ. (I) Density for the KCMF1 ZZ-DZB region (residues 1-126), shown at a threshold of 7σ. The model is labeled AF predicted where indicated.

Fig. S5

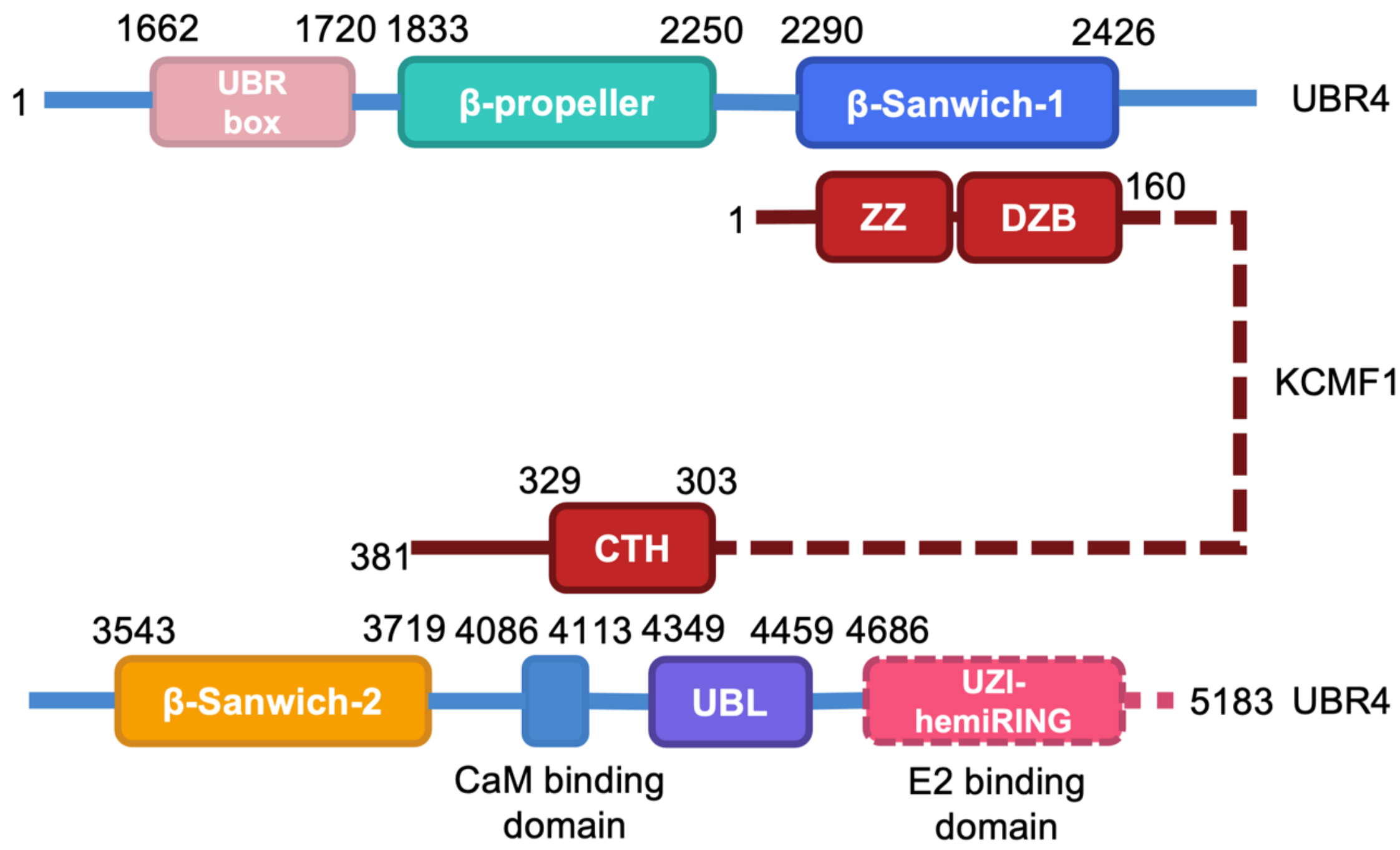

Fig. S5. Domain architecture of UBR4 and KCMF1, highlighting major motifs and interaction regions.

Fig. S6

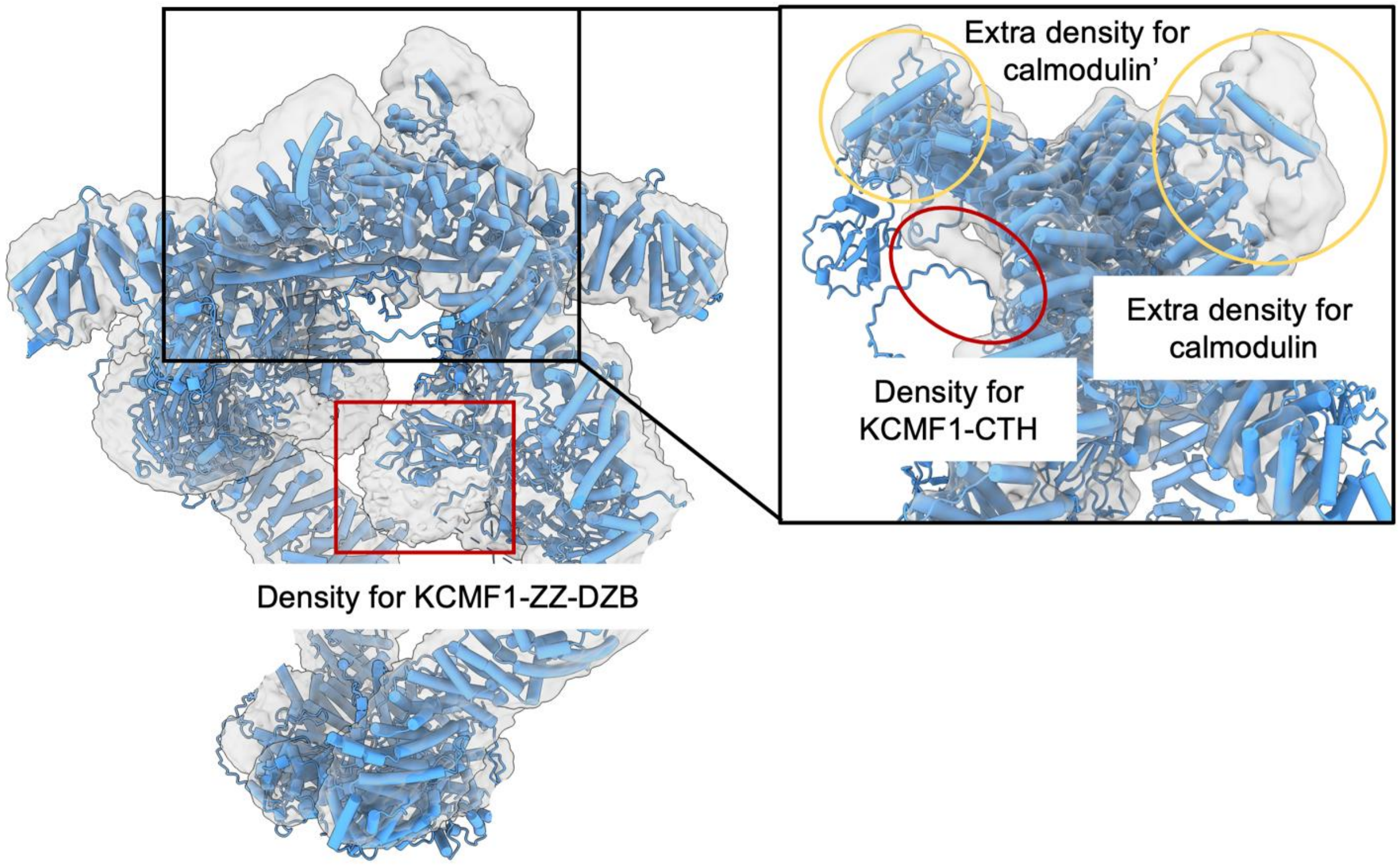

Fig. S6. Cryo-EM density for KCMF1 and calmodulin.

The overall UBR4-KCMF1-CaM complex is shown in cornflower blue, with boxed regions enlarged in the insets. The left inset highlights density for the KCMF1 ZZ-DZB domain. The right inset highlights two additional densities assigned to calmodulin and the KCMF1 C-terminal helix domain.

Fig. S7

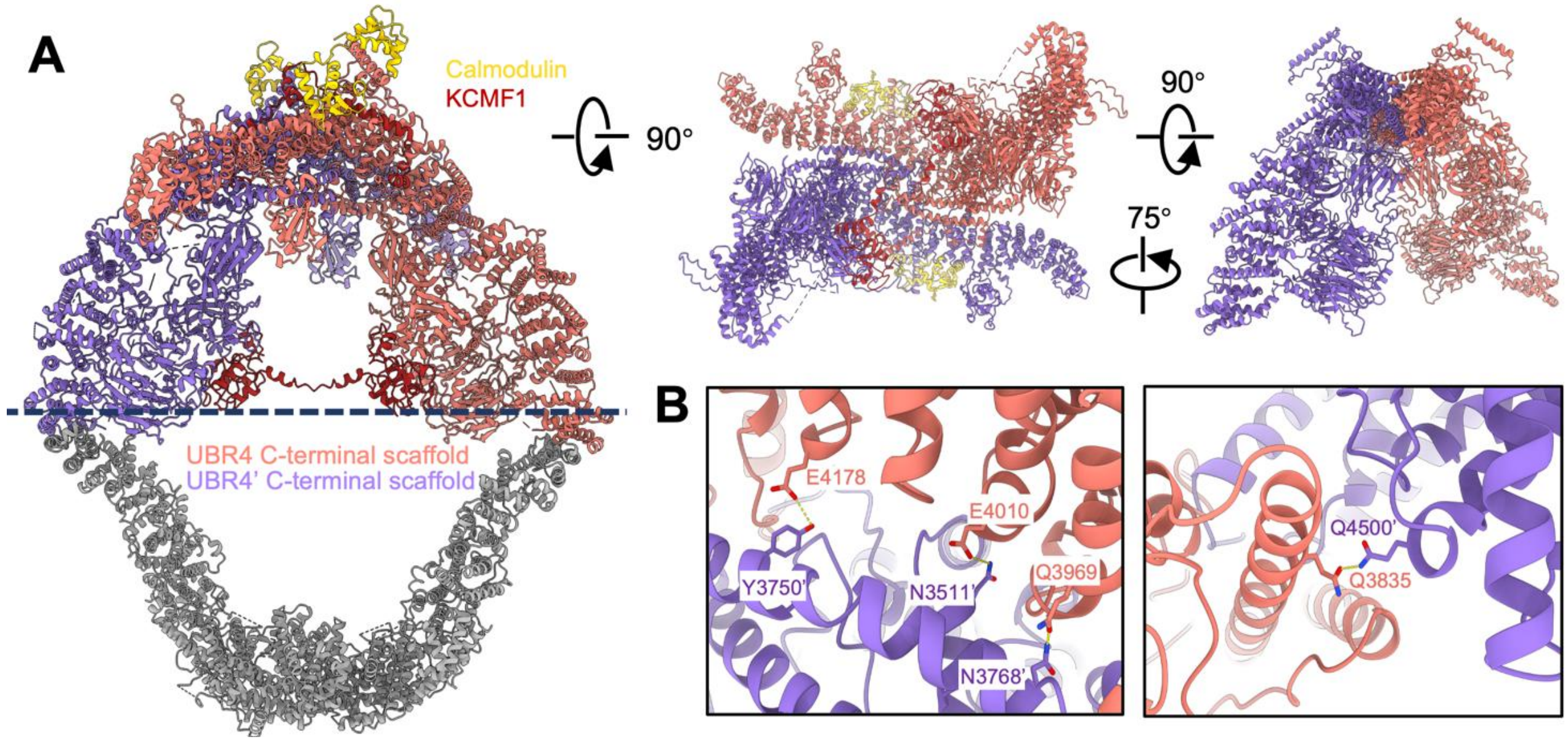

Fig. S7. Structural organization of the UBR4 C-terminal scaffold.

(A) Cryo-EM structure of the toroidal UBR4 C-terminal scaffold (residues 1663-5183) shown in two views. The left panel is rotated by 90°, and the right panel is tilted by 75°. UBR4 and UBR4' are colored salmon and medium purple, respectively.

Fig. S8

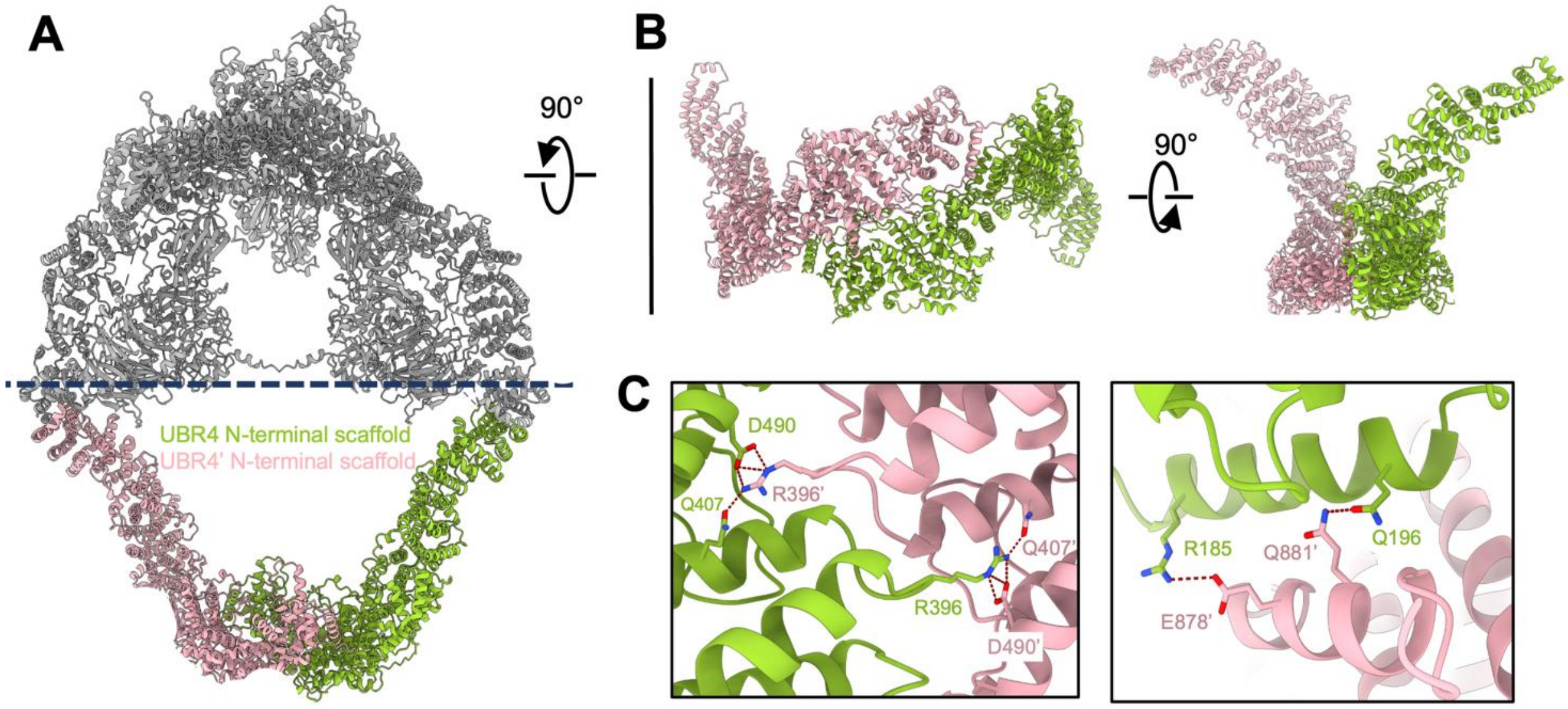

Fig. S8. Structural overview of the UBR4 N-terminal scaffold and its interaction interfaces.

(A) Overall structure of the UBR4-KCMF1-CaM complex, highlighting the N-terminal scaffolds of UBR4 (pink) and UBR4' (green). The dashed line marks the approximate boundary between the N-terminal scaffold and the rest of the complex. (B) Two orthogonal views of the UBR4 N-terminal scaffold. (C) Close-up views of interface residues. Left, R396', Q407', and D490' in UBR4' interact with R396 and D490 in UBR4. Right, E878' and Q881' in UBR4' interact with R185 and Q196 in the UBR4 N-terminal scaffold.

Fig. S9

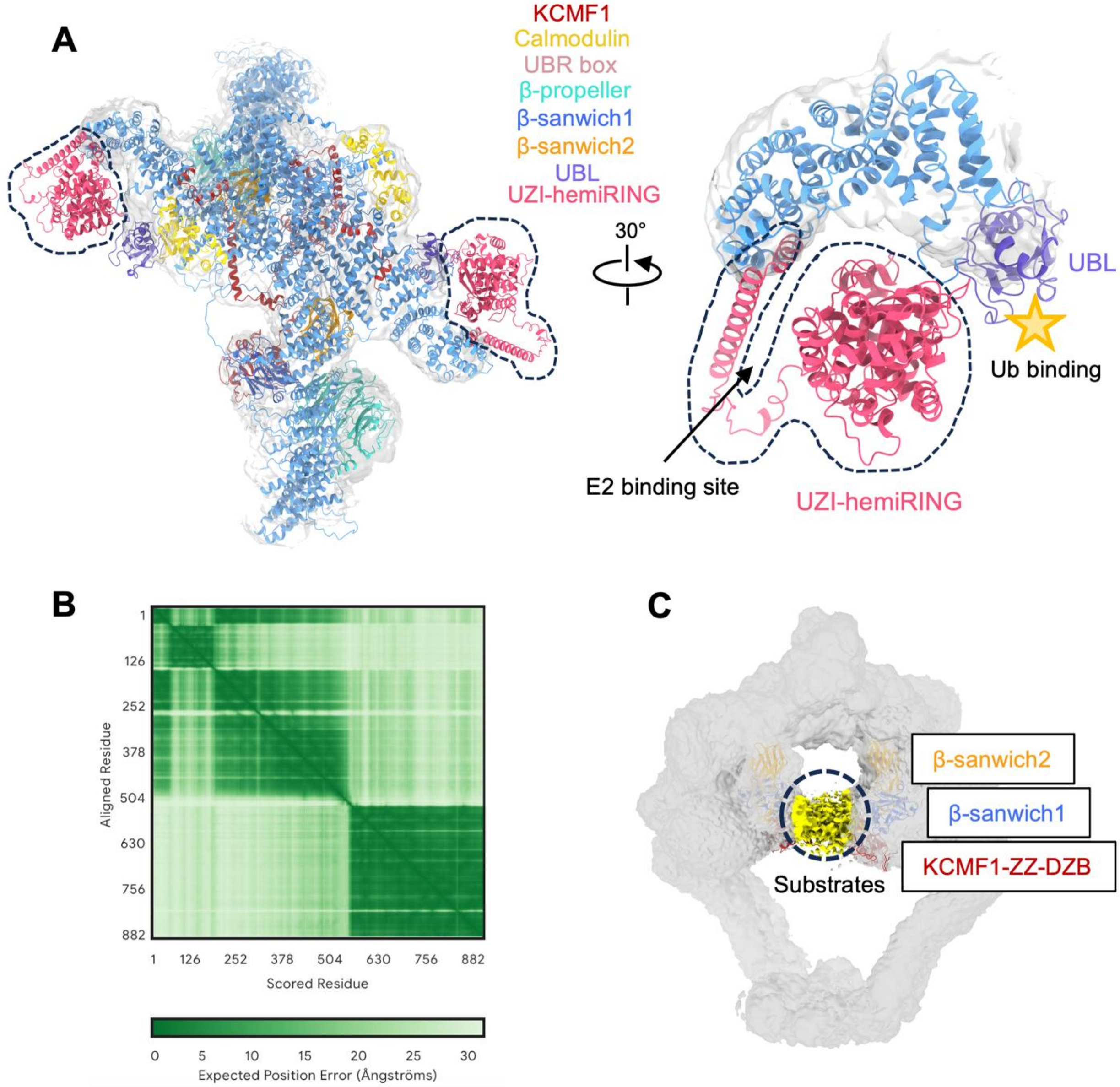

Fig. S9. Prediction of the UZI-HemiRING structure.

(A) AlphaFold 3 model of the UBR4-KCMF1-Calmodulin complex. Domains are colored as follows: calmodulin, yellow; KCMF1, red; UBR box, pink; β-propeller, turquoise; β-sandwich 1, royal blue; β-sandwich 2, orange; UZI-HemiRING, magenta. The right panel shows a 30° rotated view highlighting the putative E2-binding site and the ubiquitin-binding UBL region. (B) Heat map of the expected position error for the AlphaFold 3 model. Darker colors indicate larger predicted errors. (C) Cryo-EM density map of the UBR4-KCMF1-CaM complex, with the putative substrate region indicated by the dashed circle.

Fig. S10

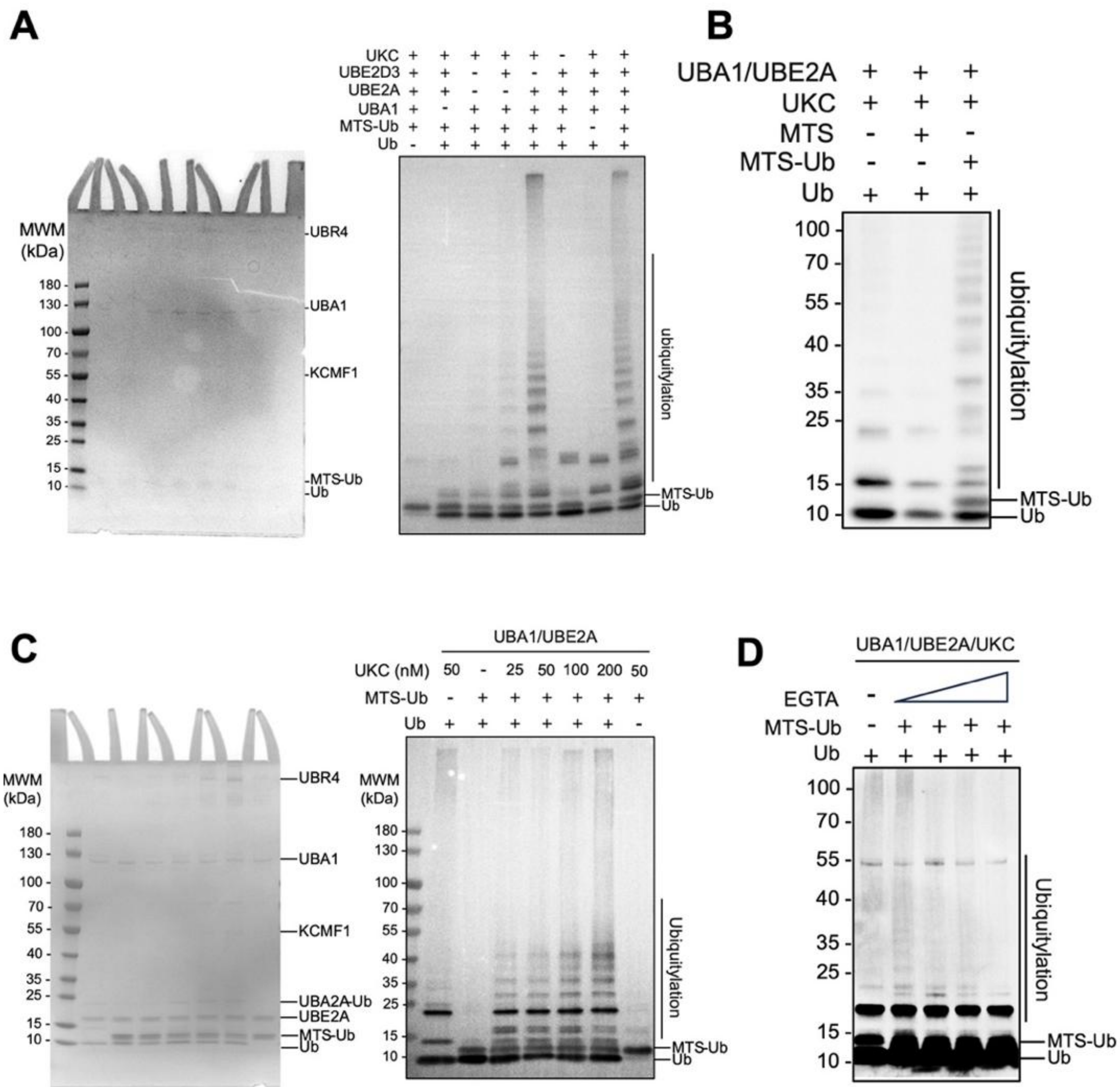

Fig. S10. Characterization of UKC-mediated ubiquitination.

(A) E2 screening for UKC-mediated ubiquitination. Left, SDS-PAGE analysis of purified assay components. Right, *in vitro* ubiquitination assays comparing UBE2D3 and UBE2A using ACOT9-MTS-Ub as substrate. (B) *In vitro* ubiquitination assays comparing ACOT9-MTS and ACOT9-MTS-Ub, showing preferential modification of the ubiquitin-fused substrate. (C) UKC concentration-dependent ubiquitination of ACOT9-MTS-Ub. Left, SDS-PAGE analysis of purified assay components. Right, *in vitro* ubiquitination assays with increasing UKC concentrations. (D) Effect of EGTA on UKC-mediated ACOT9-MTS-Ub ubiquitination. UKC, UBR4-KCMF1-CaM complex; MTS, mitochondrial targeting sequence; MTS-Ub, mitochondrial targeting sequence fused to ubiquitin; Ub, ubiquitin.

Fig. S11

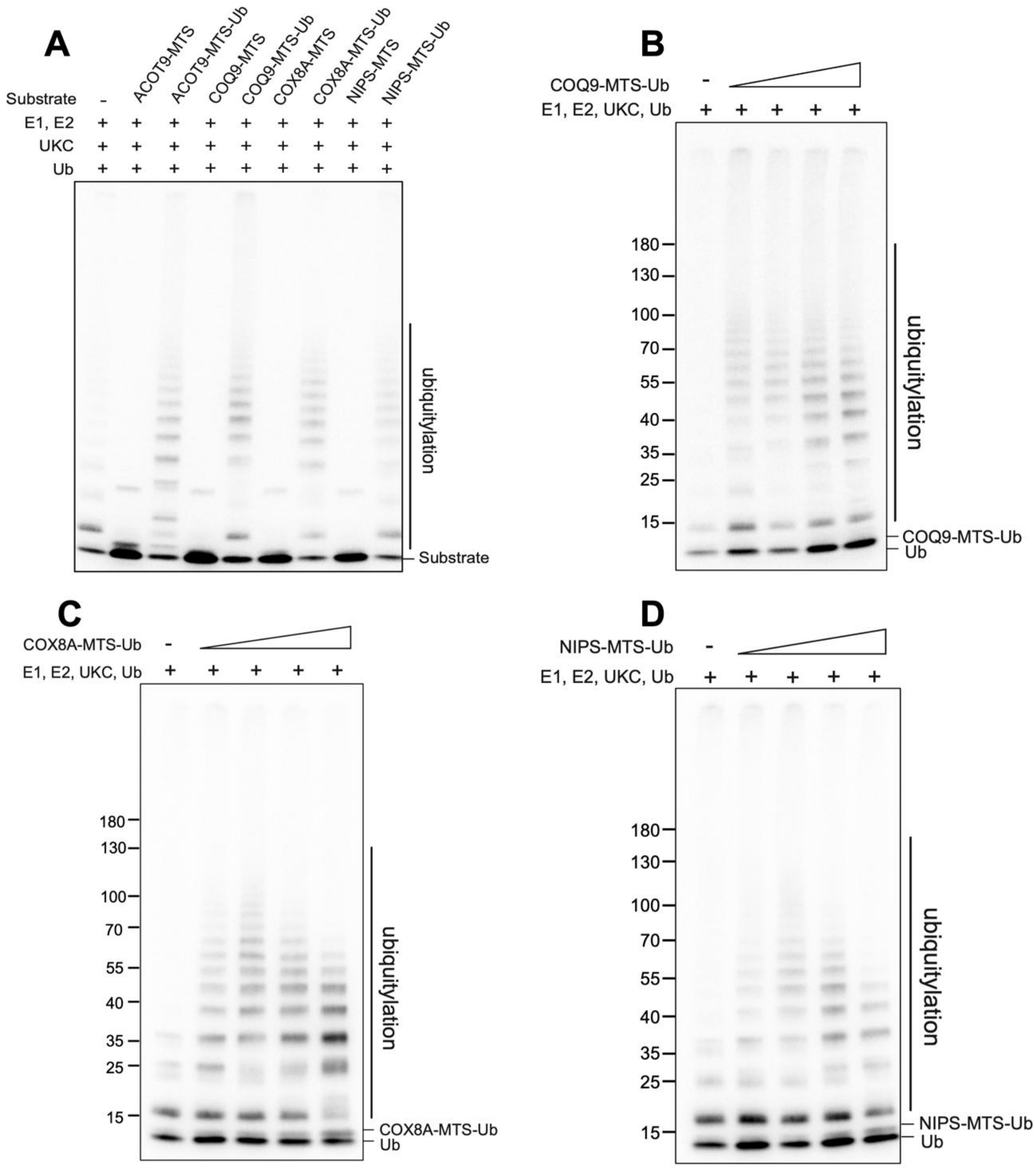

Fig. S11. UKC-mediated ubiquitination of MTS-containing substrates.

(A) *In vitro* ubiquitination assays of MTS-containing substrates, including ACOT9-MTS, COQ9-MTS, COX8A-MTS, and NIPS-MTS, and their ubiquitin-fused forms. (B-D) Substrate titration assays showing UKC-mediated ubiquitination of COQ9-MTS-Ub (B), COX8A-MTS-Ub (C), and NIPS-MTS-Ub (D). UKC, UBR4-KCMF1-CaM complex; MTS, mitochondrial targeting sequence; MTS-Ub, mitochondrial targeting sequence fused to ubiquitin; Ub, ubiquitin. NIPS-MTS/NIPS-MTS-Ub, derived from NIPSNAP1.

Fig. S12

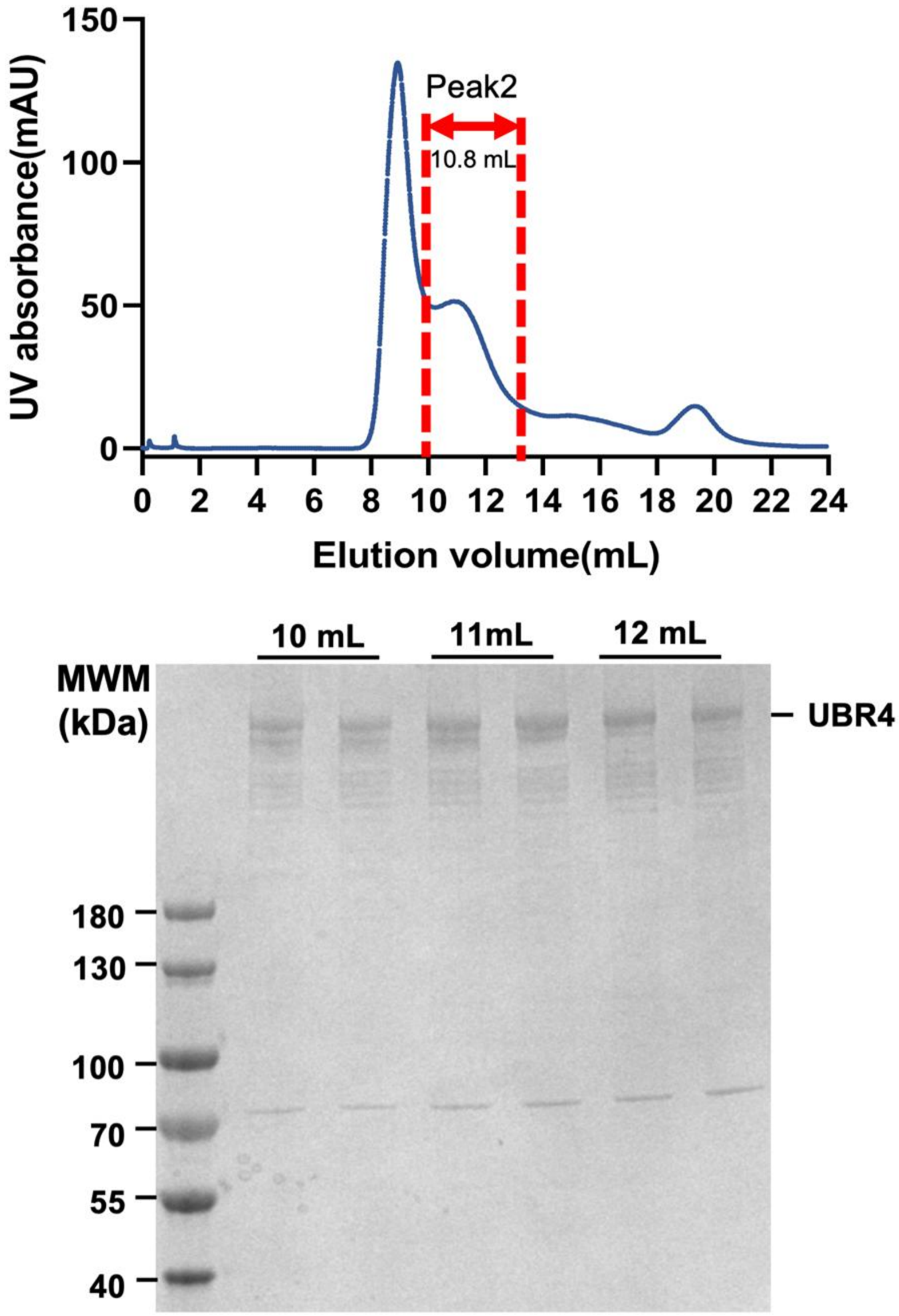

Fig. S12. Purification of UBR4 without KCMF1.

Top, UV absorbance profile from size-exclusion chromatography. The major UBR4 peak elutes at approximately 10.8 mL (peak 2; red dashed lines). Bottom, SDS-PAGE analysis of fractions collected at 10, 11, and 12 mL. MWM, molecular weight marker.

Fig. S13

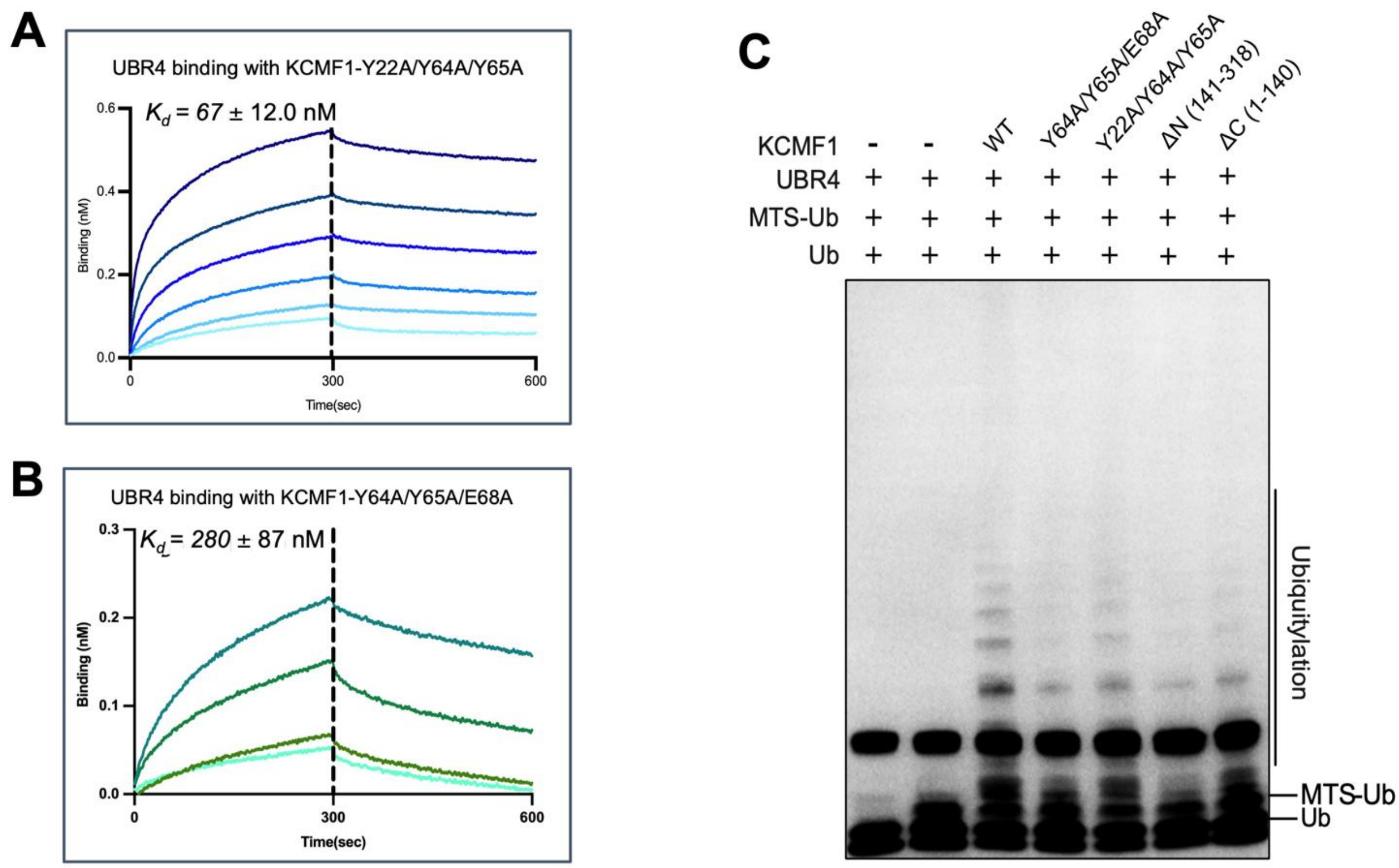

Fig. S13. Functional analysis of KCMF1 mutants in UBR4 binding and substrate ubiquitination. (A) and (B) Biolayer interferometry analysis of binding between UBR4 and KCMF1-Y22A/Y64A/Y65A or KCMF1-Y64A/Y65A/E68A. Dissociation constants (K_d) are indicated. (C) *In vitro* ubiquitination assays comparing wild-type (WT) KCMF1 and the indicated KCMF1 mutants or truncations in reactions containing UBR4, ACOT9-MTS-Ub, and ubiquitin. MTS-Ub, mitochondrial targeting sequence-tagged ubiquitin; Ub, ubiquitin.

Fig. S14

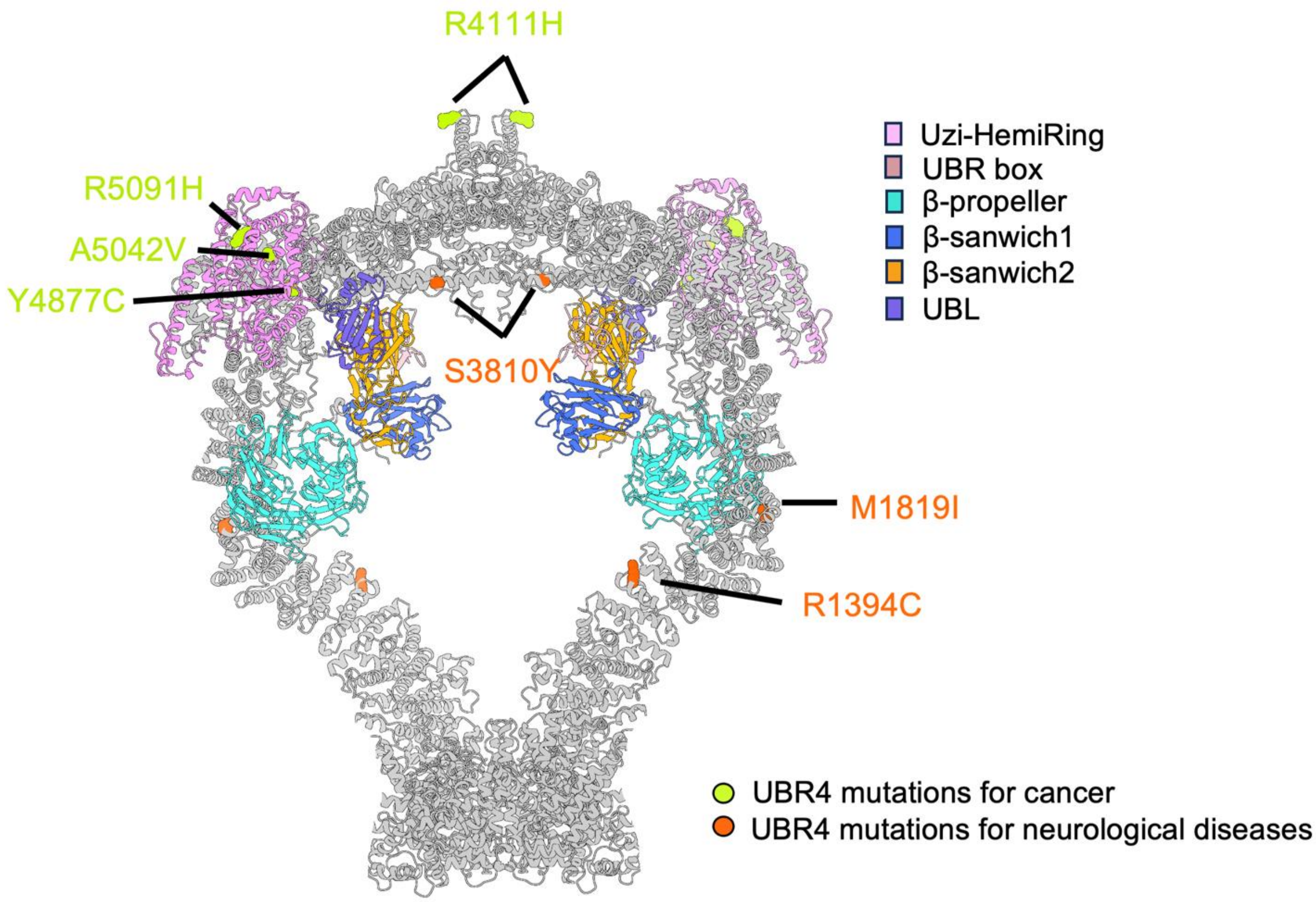

Fig. S14. Structural model of UBR4 variants associated with neurological disease and cancer. Disease-associated residues are mapped onto the UBR4-KCMF1-CaM structure. Domains are colored as follows: UZI-HemiRING, magenta; UBR box, pink; beta-propeller, turquoise; beta-sandwich 1, royal blue; beta-sandwich 2, orange; UBL, purple. Variants associated with neurological disease (R5091H, A5042V, Y4877C, R4111H, and S3810Y) are shown in green, whereas variants identified in cancer samples (M1819I and R1394C) are shown in orange.

Table S1

Mass spectrometry identification of the core components in the UBR4-KCMF1 complex.

<table>
<tr><th>Top</th><th>Accession</th><th>Gene</th><th>Area</th><th>-10LgP</th><th>Coverage(%)</th><th>Coverage(%)</th><th>#Peptides</th><th>#Unique</th><th>#Spec</th><th>Average Mass</th></tr>
<tr><td>TRUE</td><td>sp|Q5T4S7|UBR4_HUMAN</td><td>UBR4</td><td>9.77E+06</td><td>282.18085</td><td>40.46</td><td>40.46</td><td>240</td><td>239</td><td>1261</td><td>573841</td></tr>
<tr><td>TRUE</td><td>sp|P0DMV8|HS71A_HUMAN</td><td>HSPA1A</td><td>1.13E+06</td><td>199.83434</td><td>61.62</td><td>61.62</td><td>42</td><td>19</td><td>287</td><td>70052.23</td></tr>
<tr><td>TRUE</td><td>sp|P0DMV9|HS71B_HUMAN</td><td>HSPA1B</td><td>1.13E+06</td><td>199.83434</td><td>61.62</td><td>61.62</td><td>42</td><td>19</td><td>287</td><td>70052.23</td></tr>
<tr><td>TRUE</td><td>sp|P02768|ALBU_HUMAN</td><td>ALB</td><td>6.60E+05</td><td>199.70412</td><td>69.62</td><td>69.62</td><td>42</td><td>42</td><td>172</td><td>69366.68</td></tr>
<tr><td>TRUE</td><td>sp|Q9P0J7|KCMF1_HUMAN</td><td>KCMF1</td><td>3.96E+05</td><td>152.37695</td><td>33.33</td><td>33.33</td><td>16</td><td>16</td><td>91</td><td>41945.367</td></tr>
<tr><td>TRUE</td><td>sp|P35579|MYH9_HUMAN</td><td>MYH9</td><td>3.84E+05</td><td>244.3671</td><td>46.33</td><td>46.33</td><td>111</td><td>85</td><td>260</td><td>226532.23</td></tr>
<tr><td>TRUE</td><td>sp|Q9NUJ1|ABHDA_HUMAN</td><td>ABHD10</td><td>1.95E+05</td><td>155.95348</td><td>48.69</td><td>48.69</td><td>15</td><td>15</td><td>59</td><td>33932.508</td></tr>
<tr><td>TRUE</td><td>sp|P27708|PYR1_HUMAN</td><td>CAD</td><td>1.76E+05</td><td>220.82445</td><td>30.61</td><td>30.61</td><td>68</td><td>68</td><td>147</td><td>242983.72</td></tr>
<tr><td>TRUE</td><td>sp|P0DP23|CALM1_HUMAN</td><td>CALM1</td><td>1.76E+05</td><td>131.0938</td><td>51.68</td><td>51.68</td><td>9</td><td>9</td><td>60</td><td>16837.582</td></tr>
<tr><td>TRUE</td><td>sp|P0DP24|CALM2_HUMAN</td><td>CALM2</td><td>1.76E+05</td><td>131.0938</td><td>51.68</td><td>51.68</td><td>9</td><td>9</td><td>60</td><td>16837.582</td></tr>
<tr><td>TRUE</td><td>sp|P0DP25|CALM3_HUMAN</td><td>CALM3</td><td>1.76E+05</td><td>131.0938</td><td>51.68</td><td>51.68</td><td>9</td><td>9</td><td>60</td><td>16837.582</td></tr>
<tr><td>TRUE</td><td>sp|P07910|HNRPC_HUMAN</td><td>HNRNPC</td><td>1.64E+05</td><td>155.48666</td><td>40.85</td><td>40.85</td><td>17</td><td>14</td><td>51</td><td>33670</td></tr>
<tr><td>TRUE</td><td>sp|P0CG48|UBC_HUMAN</td><td>UBC</td><td>1.43E+05</td><td>116.8813</td><td>8.61</td><td>8.61</td><td>7</td><td>7</td><td>39</td><td>77038.61</td></tr>
<tr><td>TRUE</td><td>sp|P62987|RL40_HUMAN</td><td>UBA52</td><td>1.43E+05</td><td>116.8813</td><td>46.09</td><td>46.09</td><td>7</td><td>7</td><td>39</td><td>14728.311</td></tr>
<tr><td>TRUE</td><td>sp|P62979|RS27A_HUMAN</td><td>RPS27A</td><td>1.43E+05</td><td>116.8813</td><td>37.82</td><td>37.82</td><td>7</td><td>7</td><td>39</td><td>17964.914</td></tr>
<tr><td>TRUE</td><td>sp|P0CG47|UBB_HUMAN</td><td>UBB</td><td>1.43E+05</td><td>116.8813</td><td>25.76</td><td>25.76</td><td>7</td><td>7</td><td>39</td><td>25761.639</td></tr>
<tr><td>FALSE</td><td>sp|Q8TDY2|RBCC1_HUMAN</td><td>RB1CC1</td><td>1.06E+05</td><td>29.614607</td><td>0.38</td><td>0.38</td><td>1</td><td>1</td><td>9</td><td>183090.86</td></tr>
<tr><td>TRUE</td><td>sp|Q14204|DYHC1_HUMAN</td><td>DYNC1H1</td><td>8.93E+04</td><td>242.36014</td><td>25.87</td><td>25.87</td><td>113</td><td>113</td><td>163</td><td>532407.7</td></tr>
<tr><td>TRUE</td><td>sp|Q00839|HNRPU_HUMAN</td><td>HNRNPU</td><td>8.05E+04</td><td>171.7979</td><td>25.7</td><td>25.7</td><td>26</td><td>25</td><td>48</td><td>90584.43</td></tr>
</table>

Table S2

Cryo-EM data collection, refinement and validation statistics

	UKC complex N-lobe (Focus)	UKC complex C-lobe (Focus)	UKC complex (Whole)
**Data collection and processing**			
**Microscope**	Titan Krios (Thermo Fisher Scientific)	Titan Krios (Thermo Fisher Scientific)	Titan Krios (Thermo Fisher Scientific)
**Voltage (kV)**	300	300	300
**Detector**	Gatan K3 Summit	Gatan K3 Summit	Gatan K3 Summit
**Energy filter**	Gatan GIF Quantum, 20 eV slit	Gatan GIF Quantum, 20 eV slit	Gatan GIF Quantum, 20 eV slit
**Electron exposure (e^-/Å²)**	50	50	50
**Defocus range (μm)**	-1.4 to -1.8	-1.4 to -1.8	-1.4 to -1.8
**Physical pixel size (Å/pixel)**	0.827	0.827	0.827
**Data collection mode**	—	—	—
**Symmetry imposed**	C1	C1	C1
**Initial micrographs (n)**	121,111	121,111	121,111
**3D reconstruction and map statistics**			
**Software**	cryoSPARC v4.4.1	cryoSPARC v4.4.1	cryoSPARC v4.4.1
**Final particle images (n)**	244,291	244,291	244,291
**Map resolution (Å)**	3.47	3.73	3.89
**FSC threshold for resolution**	0.143	0.143	0.143
**Map sharpening B-factor (Å²)**	-90	-90	-90
**Refinement and model composition**			
**Refinement software**	Phenix 1.20.1-4487	Phenix 1.20.1-4487	Phenix 1.20.1-4487
**Ligands / nucleotides / ions**	—	4 Ca^{2+}	—
**Protein residues (modeled)**	2740	3356	9093
**Side chains assigned**	2740	3356	9093
**CC (volume)**	0.55	0.32	0.31
**CC (mask)**	0.54	0.32	0.31
**Average B factor (Å²)**	67.8	135.9	153.4
**Model validation**			
**R.m.s. deviation - bond length (Å)**	0.012	0.012	0.011
**R.m.s. deviation - bond angle (°)**	0.648	1.627	1.617
**MolProbity score**	1.92	2.70	2.42
**Ramachandran favored (%)**	96.97	95.91	96.42
**Ramachandran allowed (%)**	3.03	3.41	3.10
**Ramachandran outliers (%)**	0.00	0.68	0.48

[a]Gold-standard FSC 0.143 cutoff criteria.

Table S3

Selected for the ubiquitination assay and corresponding MTS sequences [7]

Protein	MTS sequence
ACOT9-MTS	RRAALRLCALGKGQLTPGR
COQ9-MTS	RAGWRLLQLRCLPVARCRQA
COX8A-MTS	SVLTPLLLRGLTGSARRL
NIPSNAP1-MTS	APRLCSISVTARRL

Supplementary References

1 A. Punjani, J. L. Rubinstein, D. J. Fleet and M. A. Brubaker. cryoSPARC: algorithms for rapid unsupervised cryo-EM structure determination. Nat Methods 14, 290-296 (2017).

2 A. Punjani and D. J. Fleet. 3DFlex: determining structure and motion of flexible proteins from cryo-EM. Nat Methods 20, 860-870 (2023).

3 M. van Heel and M. Schatz. Fourier shell correlation threshold criteria. J Struct Biol 151, 250-262 (2005).

4 M. Beckers and C. Sachse. Permutation testing of Fourier shell correlation for resolution estimation of cryo-EM maps. J Struct Biol 212, 107579 (2020).

5 P. Emsley, B. Lohkamp, W. G. Scott and K. Cowtan. Features and development of Coot. Acta Crystallogr D Biol Crystallogr 66, 486-501 (2010).

6 P. D. Adams, *et al.* PHENIX: a comprehensive Python-based system for macromolecular structure solution. Acta Crystallogr D Biol Crystallogr 66, 213-221 (2010).

7 D. L. Haakonsen, *et al.* Stress response silencing by an E3 ligase mutated in neurodegeneration. Nature 626, 874-880 (2024).
